# Bioactivity of *Cyperus amuricus* extracts against hepatocellular carcinoma and molecular docking analysis targeting the PI3K/AKT/mTOR pathway

**DOI:** 10.1371/journal.pone.0340868

**Published:** 2026-01-09

**Authors:** Thanh Luan Nguyen, Thanh Khoi Tu, Thien-Vy Phan, Chanh M. Nguyen, Khoa D. Nguyen, Minh Quan Pham, Hai Ha Pham Thi

**Affiliations:** 1 HUTECH Institute of Applied Science, HUTECH University, Ho Chi Minh City, Vietnam; 2 Center for Hi-Tech Development, Nguyen Tat Thanh University, Saigon Hi-Tech Park, Ho Chi Minh City, Vietnam; 3 NTT Hi-Tech Institute, Nguyen Tat Thanh University, Ho Chi Minh City, Vietnam; 4 Faculty of Pharmacy, Nguyen Tat Thanh University, Ho Chi Minh City, Vietnam; 5 Institute of Applied Science and Technology, Van Lang School of Technology, Van Lang University, Ho Chi Minh City, Vietnam; 6 Faculty of Applied Technology, Van Lang School of Technology, Van Lang University, Ho Chi Minh City, Vietnam; 7 Institute of Natural Products Chemistry, Vietnam Academy of Science and Technology, Hanoi, Vietnam; 8 Graduate University of Science and Technology, Vietnam Academy of Science and Technology (VAST), Hanoi, Vietnam; Goa College of Pharmacy, INDIA

## Abstract

*Cyperus amuricus* (Cyperaceae) has exhibited potential anticancer activity against hepatocellular carcinoma (HCC), yet its molecular mechanisms and phytoconstituent interactions with oncogenic pathways remain underexplored. This study integrates *in vitro* cytotoxicity assays and molecular docking to evaluate the effects of *C. amuricus* fractionated extracts on HCC, focusing on PI3K/AKT/mTOR signaling axis. The ethyl acetate (EA) fraction selectively inhibited HepG2 cell proliferation (IC_50_ = 159.76 µg/mL) with minimal toxicity to normal fibroblasts. Apoptotic features—cell shrinkage, membrane blebbing, nuclear condensation, and DNA fragmentation—were confirmed through DAPI staining and gel electrophoresis. Western blot analysis revealed dose-dependent suppression of phosphorylated Akt and p70S6K, indicating pathway inhibition. Molecular docking identified strong binding affinities between Cyperaceae-derived compounds and PI3K/AKT/mTOR targets, with luteolin 7-O-β-D-glucuronopyranoside-6″-methyl ester blocked PI3K activation, vitexin bound AKT’s allosteric site, and digitoxin targeted mTOR’s ATP-binding pocket, showing comparable binding energies to reference ligands. These findings suggest *C. amuricus* as a promising candidate for natural product-based HCC therapy.

## Introduction

Cancer remains a leading causes of death worldwide, with an estimated 19.98 million new cases and 9.74 million deaths reported in 2022 [1]. By 2050, annual cases are projected to reach 35.3 million, and deaths to rise to 18.5 million [2]. Hepatocellular carcinoma (HCC), or liver cancer, ranks sixth in global cancer incidence, (866,000 cases) yet third in cancer-related mortality, accounting for 758,725 deaths [1]. The high mortality is largely attributed to cirrhosis, particularly in regions such as Southeast Asia, where hepatitis is highly prevalent [3]. Despite advancements in understanding hepatocellular carcinoma (HCC), there remains a need for effective therapies. Current treatment options—including chemotherapy, radiation, and chemically derived drugs—often face significant limitations, such as drug resistance, adverse side effects, and high costs [4,5]. Therefore, exploring alternative therapeutic strategies is essential to overcome these challenges and improve patient outcomes.

The phosphatidylinositol 3-kinase/protein kinase B/mammalian target of rapamycin (PI3K/AKT/mTOR) pathway is a critical regulator of cellular processes, including metabolism, transcription, protein synthesis, cell growth, proliferation, and apoptosis [6]. PI3Ks, activated by growth factors and oncogenes, convert phosphatidylinositol-4,5-bisphosphate (PIP2) into phosphatidylinositol-3,4,5-triphosphate (PIP3), which triggers Akt activation and subsequently mTOR signaling—a pathway central to protein synthesis, cell growth [7]. Genetic mutations and dysregulation within this signaling network drive tumor progression through a cascade of biological events [8–10]. In HCC, common genetic mutations include PIK3CA (3%), PIK3R1 (1.2%), PIK3R2 (1.5%), AKT1 (0.7%), and AKT2 (1.1%) [6]. Therefore, PI3K/AKT/mTOR inhibitors represent promising small-molecule therapeutics for HCC treatment.

Molecular docking is a powerful computational tool used to predict molecular interactions, elucidate mechanisms of action, and identify potential drug candidates [11]. This technique has enabled the characterization of conformational differences between wild-type (*PI3KR* wt) and the *H1047R* mutant, facilitating the design of ligands with activity against both isoforms [12]. Docking tools have also been used to assess the selectivity of 147 ligands across four PI3K isoforms, revealing key residues responsible for subtype-specific binding [13]. Additional studies have also identified the molecular interactions of ARQ 092, a selective allosteric AKT kinase inhibitor, with AKT isoforms [14], and elucidated the binding mode of MK-2206 with human AKT isoforms [15]. Furthermore, cascaded *in silico* screening models have been integrated to discovery of 15 novel mTOR inhibitors [16]. Docking analyses of PI3K and mTOR crystal structures have revealed key active-site residues essential for dual PI3K/mTOR inhibitors binding [17].

Flavonoids are promising natural inhibitors of the PI3K/AKT/mTOR signaling pathway, exerting anticancer effects by disrupting activation and tumor-promoting processes. This inhibition induces cell cycle arrest, apoptosis induction, and angiogenesis suppression, contributing to tumor growth suppression [18]. Flavonoids are characterized by a 15-carbon backbone comprising two six-carbon benzene rings connected by a three-carbon bridge. They interact with protein residues through hydrogen bonds and hydrophobic interactions, enhancing biological activity and binding affinity [19]. Several flavonoids demonstrate strong anticancer properties, including quercetin, which induces autophagy and apoptosis in hepatocellular carcinoma; epigallocatechin gallate (EGCG), which downregulates the Akt/mTOR pathway in gastric carcinoma; and genistein, which targets the Akt and NF-κB signaling pathways in hepatocellular carcinoma [18].

*Cyperus amuricus* (*C. amuricus*), a species of the Cyperaceae family, has long been used in traditional medicine for its anti-urolithiasis and chemopreventive properties [20]. Previous research identified three antioxidant phenolic compounds—3,4-dimethoxybenzoic acid, 4-hydroxybenzoic acid, and piceatannol—with strong free radical scavenging activity [21], and its steam distillate inhibited pancreatic lipase by 57.3% *in vitro* [22]. Other Cyperaceae species, such as *C. rotundus* are rich in phenolics and flavonoids with strong anti-inflammatory properties [23]. Additionally, GC-MS analysis of *C. alternifolius* revealed a diverse phytochemical profile, including digitoxin, suggesting the presence of cardiac glycoside-like compounds within the genus [24]. Essential oils from Cyperaceae plants have also shown potent anticancer activity against HepG2, HCT-116, MCF-7, and HeLa cells through apoptosis induction and DNA fragmentation [25]. Our previous studies also demonstrated the anticancer effects of *C. amuricus* extracts on Hep3B cells via activation of the mitochondrial-dependent apoptosis pathway [26]. These initial results highlight its therapeutic potential for liver cancer. However, the mechanisms of *C. amuricus* and the molecular interactions of its known phytoconstituents with PI3K/AKT/mTOR pathways remain limited. This study investigate the bioactivity of *C. amuricus* fractional extracts using both *in vitro* cytotoxicity assays and *in silico* molecular docking, focusing on the key proteins of PI3K/AKT/mTOR signaling axis. These findings aim to expand the current understanding of this underutilized plant species and support its potential role in natural product-based therapies for HCC.

## Materials and methods

### *Cyperus amuricus* plant

No specific permits were required for the collection of *C. amuricus*, as it was gathered from non-protected areas and did not involve regulated species. *C. amuricus* specimens were collected in Tra Vinh province, Vietnam (9° 59′ 96 41″ N, 106° 12′ 53″ S), between February and June 2023. The plant collection adhered to local biodiversity regulations and ethical guidelines. The species were identified according to the internal protocol by the Department of Ecology and Evolutionary Biology. Voucher specimens (No. 06–2023/GXN) was deposited in the herbarium of University of Science, Ho Chi Minh City, Vietnam.

### Cell lines and chemicals

The Hepatocellular carcinoma cell line (HepG2, ATCC HB-8065™) and primary human dermal fibroblast (HDFa, ATCC PCS-201–012™) were obtained from the American Tissue Culture Collection (ATCC, Manassas, VA, USA). Dulbecco’s modified Eagle medium (DMEM) and penicillin-streptomycin were acquired from Cytiva, USA. Fetal bovine serum (FBS) was bought from Sigma-Aldrich, USA. Ly294002 and phosphate-buffered saline (PBS) were purchased from Thermo Fisher Scientific, USA. Thiazolyl blue tetrazolium bromide was provided by Bio Basic Inc., Canada. Methanol (MeOH), hexane (Hex), chloroform (TCM), and ethyl acetate (EA) were of analytical grade and acquired from Sigma-Aldrich. Unless otherwise stated, all other reagents were of the same grade and obtained from Sigma-Aldrich.

### Preparation of *C. amuricus* extracts

Plant specimens of *C. amuricus* were sorted, rinsed with tap water, and dried at 45–55°C until reaching constant weight. The dried plant was ground into fine powder using an electric blender, passed through a 2 mm mesh sieve, and subjected to methanolic extraction using the maceration method. The powder was immersed in methanol at a ratio of 1:7 (m/v) in a conical flask and kept at room temperature for three days; this process was repeated thrice. The mixture was then filtered through filter paper, and the solvent was evaporated under reduced pressure at 50–60°C to yield the methanol extract of *C. amuricus*. The crude extract was suspended in water and fractionated by liquid-liquid phase separation with increasing polar solvents to yield Hex, TCM, and EA fractions. Each fraction was collected, evaporated, and weighed. Yields were calculated based on the initial plant powder for the methanol extract and the crude extract for subsequent fractions. Moisture content was also recorded. The aqueous fraction yielded the highest amount, followed by MeOH, Hex, EA, and TCM (S1 Table).

### Total phenolic content

The total phenolic content (TPC) was determined using the Folin–Ciocalteu assay based on the method described by Kupina et al. [27], with slight modifications. Gallic acid (0–60 μg/mL) was used as the standard. Sample or standard solutions were mixed with 2.3 mL of distilled water, 150 μL of 10% Folin–Ciocalteu reagent, and 450 μL of 30% sodium carbonate (Na₂CO₃) solution. The mixture was vortexed and incubated in the dark for 30 minutes. Absorbance was measured at 765 nm using a spectrophotometer. TPC was calculated using the calibration curve (y = 0.0025*OD + 0.0075; R^2^ = 0.9929) and expressed as milligrams of gallic acid equivalent (GAE) per gram of extract.

### Total flavonoid content

The total flavonoid content (TFC) was determined using an aluminum chloride (AlCl_3_) colorimetric assay, following the method of Martono et al. [28], with slight modifications. Quercetin (0–60 μg/mL) was used as the standard reference. For each test, samples or standards were mixed with 800 μL of distilled water and 60 μL of 5% NaNO_2_ solution, then allowed to stand for 5 minutes. Subsequently, 60 μL of 10% AlCl_3_ solution was added and mixed thoroughly, followed by the addition of 400 μL of 1 M NaOH. After incubating for 6 minutes, 420 μL of distilled water was added, and the absorbance was measured at 510 nm using a spectrophotometer. TFC was calculated from the quercetin calibration curve (y = 0.001*OD + 0.0065; R² = 0.9955) and expressed as milligrams of quercetin equivalent (QE) per gram of extract.

### *In vitro* cytotoxicity

HepG2 and HDFa cells were cultured in DMEM medium supplemented with 10% FBS, 100 IU/mL penicillin, and 0.1 mg/mL streptomycin. Cells were maintained in a humidified incubator at 37°C with 5% CO_2_, and their morphology and confluency were observed using a light microscope.

Cytotoxicity of *C. amuricus* fractions on HepG2 and HDFa cells was evaluated using the MTT assays [29]. Cells were seeded in 96-well plates at a density of 1 × 10^4^ cells/well and incubated for 24 hours to achieve approximately 80% confluency. The test fractions were then added at a final concentration of 150 μg/mL and incubated for an additional 24 hours. Ly294002 (10 μM) served as a positive control. After treatment, the medium was replaced with 100 µL of fresh culture medium containing 20 µL of MTT solution (5 mg/mL), and cells were incubated for 4 hours. The medium was then removed, followed by the addition of DMSO (100 µL) to solubilize the formazan crystals. Absorbance was measured at 595 nm using an ELISA reader (Biotek, USA). The percentage of cell viability was calculated using the following formula:


$$\text{Cell}\;\text{viability}\;(\%)\;=\;(\frac{A_{sample}-\;A_{blank}}{A_{control}-\;A_{blank}})\text{x}\;\text{100}\%\;$$

Where A_sample_ denoted the absorbance of the tested fractions, A_blank_ represented the absorbance of the blank (medium without cultured cells), and A_control_ represented the absorbance of the untreated cells (cultured cells without extract treatment).

To determine the IC_50_ value, *C. amuricus* extracts were diluted to a series of concentrations ranging from 50 to 200 μg/mL and applied to cells. The relationship between extract concentration and cell viability was modeled using linear regression (Microsoft Excel, 2016). The IC_50_, defined as the concentration that reduces cell viability by 50%, was interpolated from the resulting regression line.

### DAPI staining

Nuclear morphology was evaluated using the DAPI (4’,6-diamidino-2-phenylindole) staining assay [30]. HepG2 cells were treated with *C. amuricus* extracts at concentrations of 100, 150, and 200 µg/mL for 24 hours. After treatment, cells were washed thrice with PBS and fixed in 4% paraformaldehyde for 15 minutes. After additional PBS washes, the fixed cells were incubated with DAPI solution (1 µg/ml in methanol) in the dark for 20 minutes. Nuclear morphology was visualized using a laser scanning confocal microscope (Carl Zeiss LSM 700, Germany). The DAPI-stained nuclei were analysed using ImageJ (version 1.52a, National Institutes of Health, USA) to automatically measure nuclear area and form factor (circularity). The nuclear area factor (NAF) was then calculated by dividing nuclear area by its circularity and used as a quantitative indicator of morphological changes associated with apoptosis [31].

### DNA fragmentation

DNA fragmentation in HepG2 cells treated with *C. amuricus* extract was assessed using gel electrophoresis [32]. Genomic DNA was isolated using a DNeasy® Blood and Tissue kit (Qiagen GmbH, Hilden, Germany). A 10 µL aliquot of purified DNA was loaded onto a 1.5% agarose gel (Life Technologies Inc., Grand Island, NY, USA) prepared in TAE buffer and electrophoresed at 100 V for 50 minutes. DNA bands was then visualized by ethidium bromide staining (Sigma-Aldrich) under a UV transilluminator (Vilber Lourmat, Marne-la-Vallée, France).

### Western bloting

HepG2 cells were washed with PBS and lysed in extraction buffer (Bio Basic Inc., Canada). Lysates were incubated on ice for 30 minutes, then centrifuged at 14,000 rpm for 20 minutes at 4°C. Supernatants containing total proteins were separated by 10% SDS-PAGE and transferred to nitrocellulose membranes (Thermo Fisher Scientific, USA). Membranes were blocked with 5% skim milk in PBST (PBS with 0.5% Tween-20) for 1 hour. Overnight incubation at 4°C was performed with primary antibodies including phospho-Akt (Ser473) (ab8932, Abcam, Cambridge, UK; 1:1000), total Akt (ab8805, Abcam; 1:1000), phospho-p70S6 Kinase (Ser371) (ab109393, Abcam, Cambridge, UK; 1:1000), total p70S6 Kinase (ab131526, Abcam, Cambridge, UK; 1:1000), and β-actin (ab8227, Abcam, Cambridge, UK; 1:5000). Following three PBST washes, membranes were incubated with HRP-conjugated secondary antibodies (anti-rabbit or anti-mouse IgG; Cell Signaling Technology) for 1 hour at room temperature. Protein bands were detected using enhanced chemiluminescence (ECL; Thermo Fisher Scientific, USA). Band densitometry was analyzed by Fiji (National Institutes of Health, USA)

### Molecular docking

#### Proteins and co-crystalized ligand preparation.

PI3K, AKT, and mTOR were selected as target receptor proteins to evaluate the anti-hepatocellular carcinoma activity of *C. amuricus*. The crystalized structures of these proteins, along with their respective co-crystallized ligands, were retrieved from the Protein Data Bank (PDB) (Table 1). Before docking, all water molecules and heteroatoms were removed to optimize binding site accessibility. Grid boxes for each protein were defined using BIOVIA Discovery Studio 2024 software (Table 1).

**Table 1:** Proteins and co-crystallized ligands used in molecular docking studies of hepatocellular cancer.

No.	Protein	PDB	Resolution	Co-crystallized ligand	Active site (X, Y, Z)	Grid resolution (Å)	Reference
1	PI3K	7K6M	2.41 Å	PF-06843195 (VXY)	−18.477, 10.726, 30.579	21	[13]
2	AKT	5KCV	2.70 Å	ARQ092 (Miransertib)	−8,846, −0,567, −17,488	15	[14]
3	mTOR	4JSV	3.50 Å	ADP (Adenosine-5’- Diphosphate)	50,124, −1,795, −45,903	16	[33]

#### Ligand and control preparation.

A selection of bioactive compounds from the Cyperaceae family, previously reported to exhibit anticancer activity, were selected through a literature search in the PubMed database. Their three-dimensional structures were generated using Avogadro software and subsequently optimized through energy minimization using the MMFF94 force field.

### Docking simulation

Before the docking simulation, polar hydrogens and Kollman charges were assigned to the receptor proteins. Molecular docking was performed using Autodock Tools to predict the top-ranked binding poses based on binding energy (∆G) between ligands and receptors, where lower ∆G values indicate stronger molecular interactions. The interactions of ligand–receptor complexes were analyzed and visualized using PyMOL software.

### Statistical analysis

All experiments were independently performed in triplicate, and the results are expressed as means ± standard deviation (SD). One-way analysis of variance (ANOVA) was conducted using Statgraphics Centurion 19 software to compare group differences. Statistical significance was determined at *P* < 0.05 using the LSD test.

## Results

The fractionated extracts of *C. amuricus* were analyzed for TPC and TFC, with concomitant cytotoxicity assessment against HepG2 and HDFa cell lines. Treatment selectively induced dose-dependent morphological alterations in HepG2, while HDFa cells remained morphologically unaffected. Apoptotic features in HepG2 cells were confirmed through DAPI staining, DNA fragmentation, and quantification of Nuclear Area Factor (NAF), which revealed significant nuclear shrinkage and loss.Western blot analysis further demonstrated a reduction in phosphorylated AKT (Ser473) and p-p70S6 Kinase (Ser 371), indicating suppression of PI3K/AKT/mTOR pathway activation. Before molecular docking, the protocol was validated through redocking of crystallized ligands, achieving strong alignment with crystallographic binding poses. Subsequent docking analysis identified key phytoconstituents of *C. amuricus* and related Cyperaceae species with high binding affinities to key components of the PI3K/AKT/ mTOR pathway.

### Total phenolic and flavonoid content of *C. amuricus* extracts

**Fig 1:**
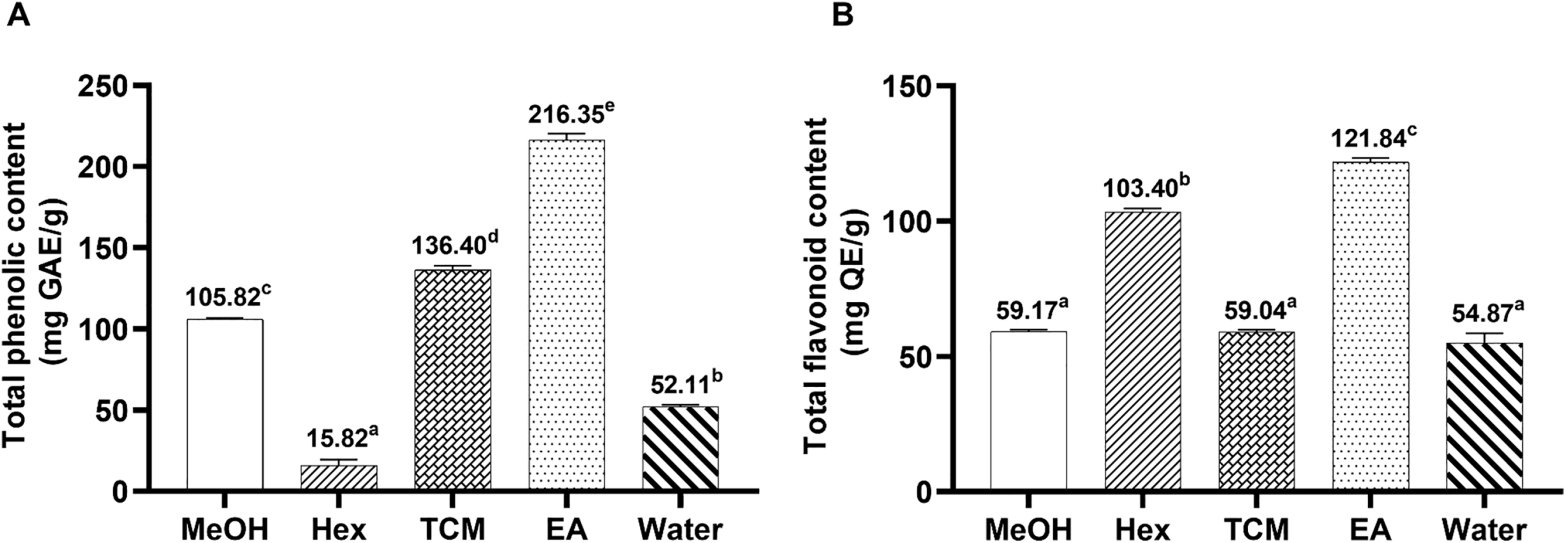
Total phenolic content (A) and flavonoid contents (B) in *C. amuricus* extracts. Abbreviations: GAE (gallic acid equivalent), QE (quercetin equivalent), and extraction solvents: Methanol (MeOH), Hexane (Hex), Chloroform (TCM), and Ethyl Acetate (EA). Letters indicate significant differences among extracts (P ≤ 0.05).

TPC and TFC, two major bioactive groups contributing to antioxidant activity and other biological effects were quantified in *C. amuricus* fractional extracts using spectrophotometric assays. TPC and TFC were statistically different between the fractions as indicated by superscript letters in Fig 1 (P ≤ 0.05). For TPC, the EA extract exhibited the highest TPC (216.35 ± 3.89 mg GAE/g extract), followed by the TCM fraction, total MeOH extract, and aqueous extract, with respective values of 136.40 ± 2.70, 105.82 ± 0.83, and 52.11 ± 1.05 mg GAE/g extract. The Hex fraction had the lowest TPC, recorded at 15.82 ± 3.70 mg GAE/g extract (Fig 1A). Regarding TFC, the EA fraction also contained the highest level (121.84 ± 1.52 mg QE/g extract), followed by the Hex fraction (103.40 ± 1.29). The MeOH, TCM, and aqueous fractions showed no significant differences, with values of 59.17 ± 0.74, 59.04 ± 0.77, and 54.87 ± 3.60 mg QE/g extract, respectively (Fig 1B).

### *C. amuricus* extracts inhibits hepatocellular carcinoma viability

**Fig 2:**
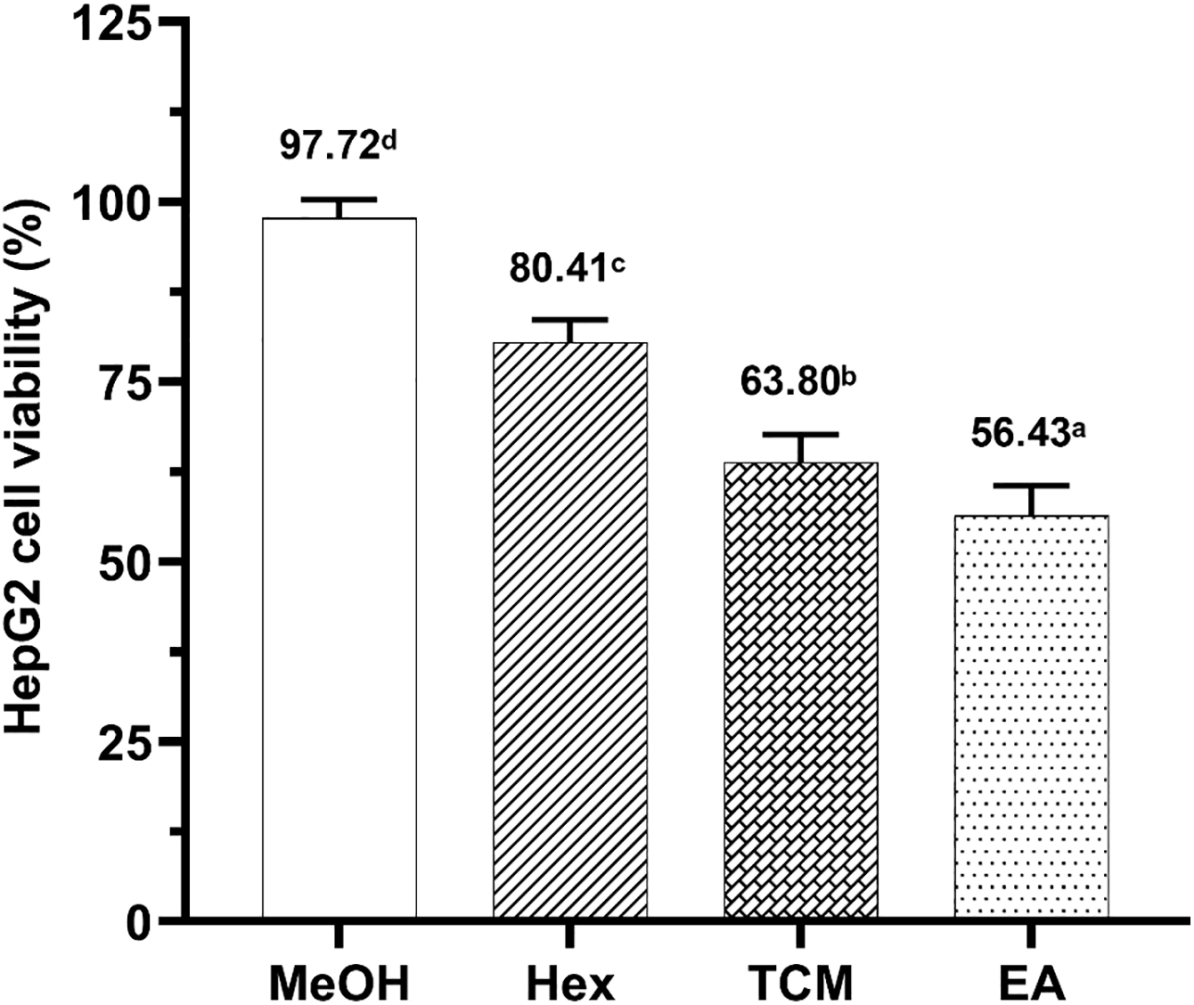
Cytotoxicity of *C. amuricus* extracts on HepG2 cells. HepG2 cells were treated with *C. amuricus* extracts, including Methanol (MeOH), Hexane (Hex), chloroform (TCM), and Ethyl acetate (EA), at 150 μg/mL, for 24 h. Cell viability was determined using the MTT assay. Superscript letters indicate statistically significant differences in means among extracts *(p < 0.05).*

The inhibitory effects of *C. amuricus* extracts on hepatocellular carcinoma HepG2 cells were evaluated by treating cells with different fractions (MeOH, Hex, TCM, and EA) at 150 μg/mL for 24 hours. Among these, the EA fraction exhibited the strongest inhibitory effect, reducing cell viability to 56.43%. In comparison, TCM, Hex, and MeOH treatment resulted in cell viability of 63.80%, 80.41%, and 97.72%, respectively (Fig 2).

### Ethyl acetate extract of *C. amuricus* selectively inhibits hepatocellular carcinoma growth

**Fig 3:**
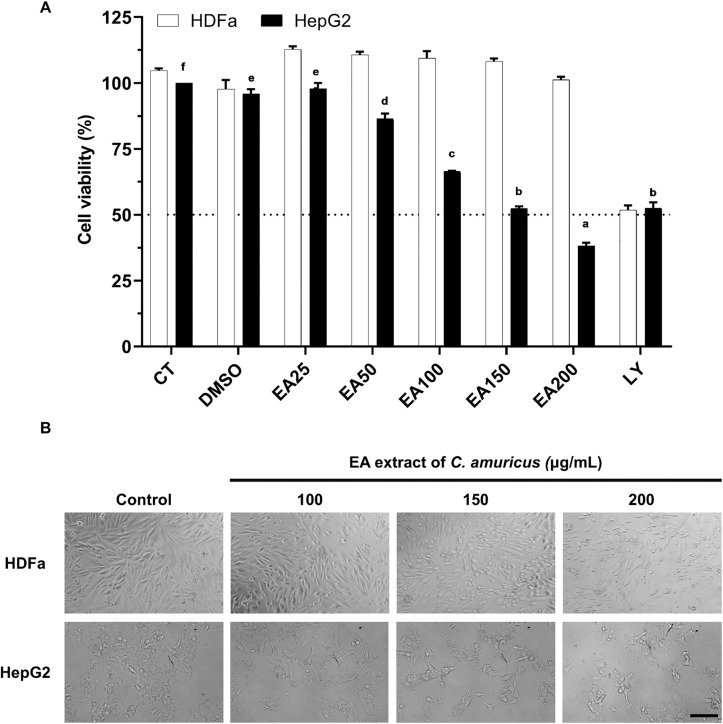
Cytotoxic effects of ethyl acetate extract of *C. amuricus* on HepG2 and HDFa cells. A. Cell viability of HepG2 and HDFa cells treated with ethyl acetate (EA) extract of *C. amuricus* for 24 h. Treatment groups include CT (untreated control), DMSO (0.05% DMSO-treated), EA20–EA200 (EA extract at 25, 50, 100, 150, and 200 μg/mL), and LY (Ly294002, 10 μM-treated). Superscript letters indicate statistically significant differences among treatment groups (p < 0.05). B. Phase-contrast images of HepG2 and HDFa cells after EA extract treatment (100, 150, 200 μg/mL, 24 h). Control: untreated; Scale bar: 100 μm.

The EA extract from *C. amuricus* was selected for further investigation due to its relatively stronger cytotoxic activity compared with other fractions. The extract induced dose-dependent cytotoxicity in HepG2 cells, while untreated and DMSO-treated cells showed no significant toxicity. At concentrations of 25–50 µg/mL, the extract caused mild effects, with cell viability maintained between 86% and 97%. However, higher concentrations (150–200 µg/mL) significantly reduced cell viability to 38–52% (p < 0.05) (Fig 3A), with an estimated IC_50_ value of 159.76 µg/mL. In contrast, the EA showed no inhibitory effects on HDFa cells, as viability remained at 101% even at 200 µg/mL. By comparison, Ly294002, a non-selective PI3K inhibitor, induced significant cytotoxicity in both HepG2 and HDFa cells. Morphological changes corresponded to cell viability results. HDFa maintained the normal morphology in the presence of the *C. amuricus* extract, while HepG2 cells displayed dose-dependent alterations. At lower concentrations, cells became rounded and detached, at 150–200 µg/mL, these changes were more pronounced, with clear shrinkage and membrane bulges or protrusions (Fig 3B).

### *C. amuricus* extract induces nuclear condensation and DNA fragmentation in HepG2 cells

**Fig 4:**
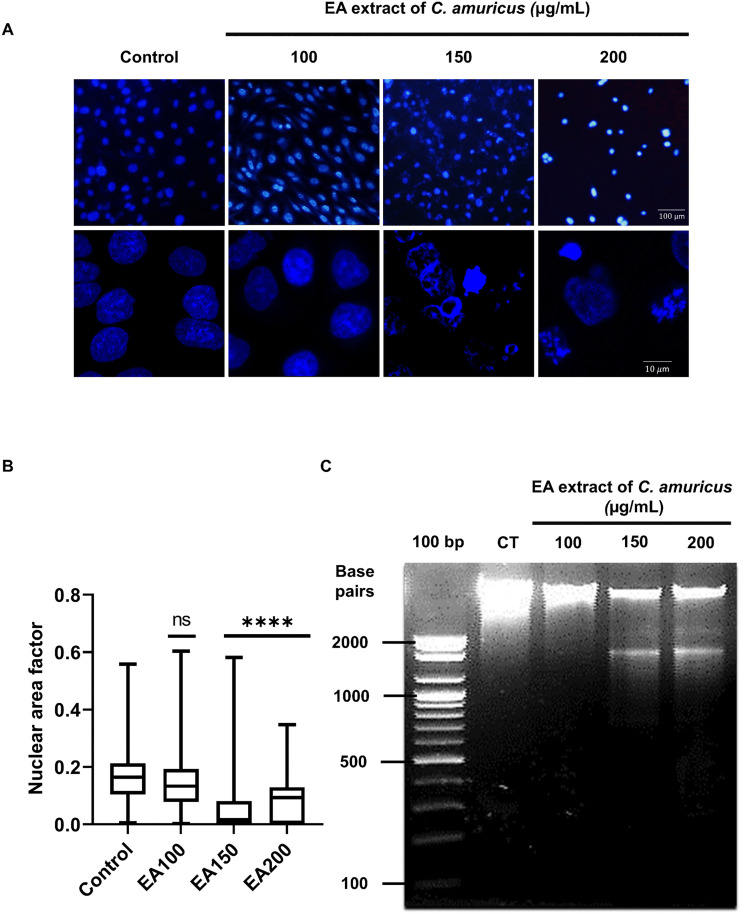
Ethyl acetate (EA) extract of *C. amuricus* induces nuclear condensation and DNA fragmentation in HepG2 cells. A. DAPI staining of HepG2 cells treated with EA extracts (100, 150, 200 μg/mL, 24 h). The top row represents images captured using a 4X objective, while the bottom row corresponds to pictures taken with a 100X objective. B. Box-and-whisker plot of nuclear area factor across control and EA treated HepG2 cells, ns: not significant. C. Agarose gel electrophoresis of DNA isolated from HepG2 cells treated with EA extracts (100, 150, 200 μg/mL, 24 h), CT (untreated control), Lane 100 bp: 100 bp DNA ladder (cat. no. D-1030; Bioneer Corporation, Daejeon, Korea) with bands at 100-2000 base pairs.

The nuclear morphology and DNA integrity of *C. amuricus*-treated HepG2 cells were examined to assess apoptotic changes. Fluorescence images revealed chromatin condensation forming dense rings along the nuclear envelope, resulting in increased fluorescence intensity compared with the untreated cells (Fig 4A). At concentrations of 150–200 µg/mL, *C. amuricus* induced nuclear fragmentation, producing multiple smaller nuclear bodies, characteristics of apoptosis [34]. Apoptosis quantification was further quantified using the NAF, a validated metric reflecting nuclear shrinkage during apoptosis (Eidet et al., 2014; Helmy & Abdel Azim, 2012). HepG2 cells treated at 150–200 µg/mL showed a pronounced reduction in NAF, whereas treatment at 100 µg/mL did not differ statistically from the untreated cells (Fig 4B). These results quantitatively support apoptotic activity of the extract and align with observed morphological alterations. DNA fragmentation, another features of apoptosis, was confirmed through gel electrophoresis. Genomic DNA purified from untreated cells displayed intact, sharp bands, whereas DNA from treated cells showed smeared patterns indicative of fragmentation. This effect was more pronounced at higher concentrations (150–200 µg/mL) (Fig 4C).

### Ethyl acetate extract of *C. amuricus* suppress AKT and p70S6 Kinase phosphorylation

**Fig 5:**
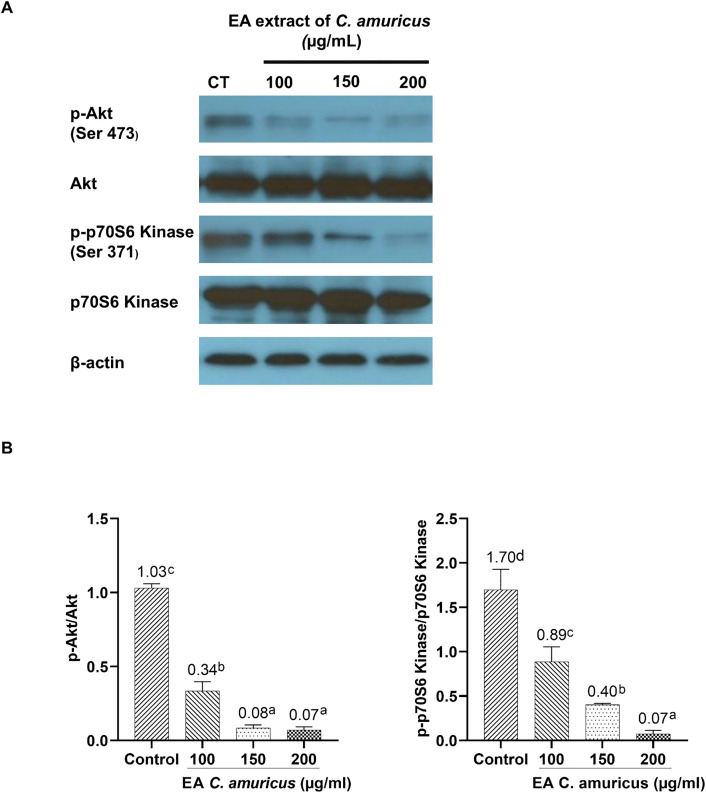
Ethyl acetate (EA) extract of *C. amuricus* suppresses phosphorylation of Akt and p70S6 kinase in HepG2 cells. A. Western blot analysis of phosphorylated Akt (p-Akt), total Akt, phosphorylated p70S6 kinase (p-p70S6 kinase), total p70S6 kinase, and β-actin in HepG2 cells treated with EA extract (100, 150, and 200 μg/mL) for 24 h. CT: untreated control. B. Quantification of p-Akt/Akt and p-p70S6 kinase/p70S6 kinase ratios. Superscript letters indicate statistically significant differences among treatment groups (p < 0.05).

Western blot analysis was performed to evaluate the effect of the EA extract of *C. amuricus* on the phosphorylation status of key signaling proteins in HepG2 cells. Cells were treated with increasing concentrations of the extract (100, 150, and 200 µg/mL), and protein expression levels were compared with untreated controls. The results showed a dose-dependent decrease in phosphorylated Akt (Ser473) and phosphorylated p70S6 Kinase (Ser371), while total Akt and total p70S6 Kinase levels remained relatively unchanged (Fig 5A). Quantitatively, the p-Akt/Akt ratio declined from approximately 1.03 in control cells to 0.34, 0.08, and 0.07 at 100, 150, and 200 µg/mL, respectively. Likewise, the p-p70S6/p70S6 ratio decreased from roughly 1.70 in controls to 0.89, 0.40, and 0.07 across the same concentration range (Fig 5B). These findings indicate that the EA extract of *C. amuricus* could suppresses PI3K/AKT/mTOR pathway activity in a concentration-dependent manner, potentially contributing to its observed anti-proliferative and pro-apoptotic effects.

### Redocking of co-crystallized ligands

Molecular docking serves as a powerful tool to predict molecular interactions, elucidate mechanisms of action, and identify potential drug leads [11]. To validate the docking protocol, co-crystallized ligands were re-docked into the active sites of their corresponding receptor proteins before simulation. The results showed that the binding energies of PI3K-VXY, AKT-ARQ092, and mTOR-ADP complexes were −9.30, −13.90, and −7.00 kcal/mol, respectively, indicating strong ligand-protein interactions. These binding affinities should function as reference standards for screening the potential of bioactive compounds derived from Cyperaceae plants. In addition, the re-docked conformations of VXY, ARQ092, and ADP in PI3K, AKT, and mTOR exhibited minimal deviations from their crystallographic poses, with root-mean-square deviations (RMSD) of 0.968, 0.289, and 1.131 Å, respectively (Table 2). The re-docking parameters were also applied to FDA-approved inhibitors targeting PI3K (alpelisib) and AKT (capivasertib). These compounds exhibited binding affinities ranging from −10.00 to −9.80 kcal/mol and RMSD values 1.779–2.901 Å (S2 Table). Collectively, these results support the validity and suitability of protocol for screening bioactive compounds.

**Table 2:** Redocking of co-crystallized ligands to receptor proteins.

Protein	Co-crystallized ligand	Types of interactions	Amino acid residues
PI3K	PF-06843195 (VXY)	Hydrogen bond	Ser774, Lys802, Asp805, Asp810, Val851, Gln859, Asp933
		Halogen bond	Val851
		π-Alkyl hydrophobic	Trp780, Val850, Ile932
		π-σ hydrophobic	Ile848
AKT	ARQ092 (Miransertib)	Hydrogen bond	Thr211, Asp274
		Carbon-hydrogen bond (C–H⋯π)	Ile290
		π-π stacking	Trp80
		π-Alkyl hydrophobic	Arg273
		π-σ hydrophobic	Leu210, Leu264, Val270
mTOR	ADP (Adenosine-5’- Diphosphate)	Hydrogen bond	Ser2165, Gln2167, Lys2187, Glu2190, Gly2238, Val2240
		π-Alkyl hydrophobic	Leu2185, Ile2356
		π-π stacking	Trp2239
		π-sulfur bond	Met2345

**Fig 6:**
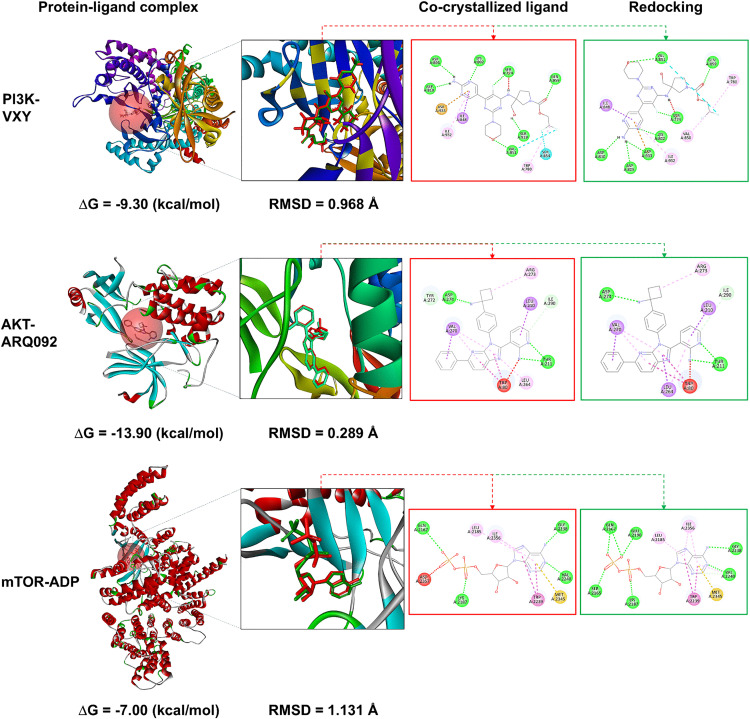
The co-crystallized ligands (VXY, ARQ092, ADP) were re-docked to PI3K, AKT, and mTOR proteins.

The molecular interactions between ligands and active-site amino acid residues were further analyzed, revealing consistent binding patterns before and after re-docking (Fig 6). VXY formed hydrogen bonds with conserved residues, including Ser774, Lys802, Asp805, Asp810, Val851, Gln859, and Asp933, which are key contributors to ligand binding and isoforms selectivity targeting PI3K [13]. Notably, VXY established a halogen bond via its fluorine group interacting with the nonpolar residue Val851. Although halogen interactions contributes less to overall binding stability, they may subtly influence the biological activity of compounds [35]. Additionally, the ligand formed hydrophobic interactions, including π-alkyl bonding with Trp780, Val850, and Ile932, as well as a π-σ bond with Ile848, contributing to the stability of the protein-ligand complex. Regarding ARQ092, this redocked ligand formed signature bonds with the binding site of AKT (Fig 6). ARQ092 established hydrogen bonds with Thr211 and Asp274 and carbon-hydrogen bond with Ile290, supporting its strong binding affinity. Additional bondings included π-π stacking with Trp80 in the pleckstrin homology domain, π-σ interactions with Val210, Leu264, and Leu210, and π-alkyl interactions with Arg273, collectively anchoring the ligand within the active site, thereby enhancing its AKT inhibitory mechanism. The highly conserved ATP-binding pocket of mTOR kinase comprises residues Ile2163, Ser2165, Lys2187, Glu2190, Tyr2225, Trp2239, Val2240, Thr2245, Asn2343, Met2345, Ile2356, and Asp2357 [36]. Molecular docking simulations showed that the co-crystallized ADP formed hydrogen bonds with Gln2167, Glu2190, Ser2165, Lys2187, Gly2238, and Val2240. Additional hydrophobic interactions were observed, including π-alkyl bonds with Leu2185 and Ile2356, and a π-sulfur bond with Met2345, all within the hydrophobic pocket. A π-π stacking interaction was also identified between the aromatic ring of ADP and the indole group of Trp2239 located within the hinge region of the mTOR protein (Fig 6). These hydrophobic interactions enhanced the stability of the protein-ligand complex. Notably, a previous study highlighted the necessity of hydrogen bonds between an inhibitor and Val2240 for mTOR inhibitory activity, while π-π stacking with Trp2239 was identified as a key factor of the potency and specificity of mTOR inhibitors [16].

### The molecular docking analysis of phytochemicals from the Cyperaceae family with the PI3K/AKT/mTOR protein

**Fig 7:**
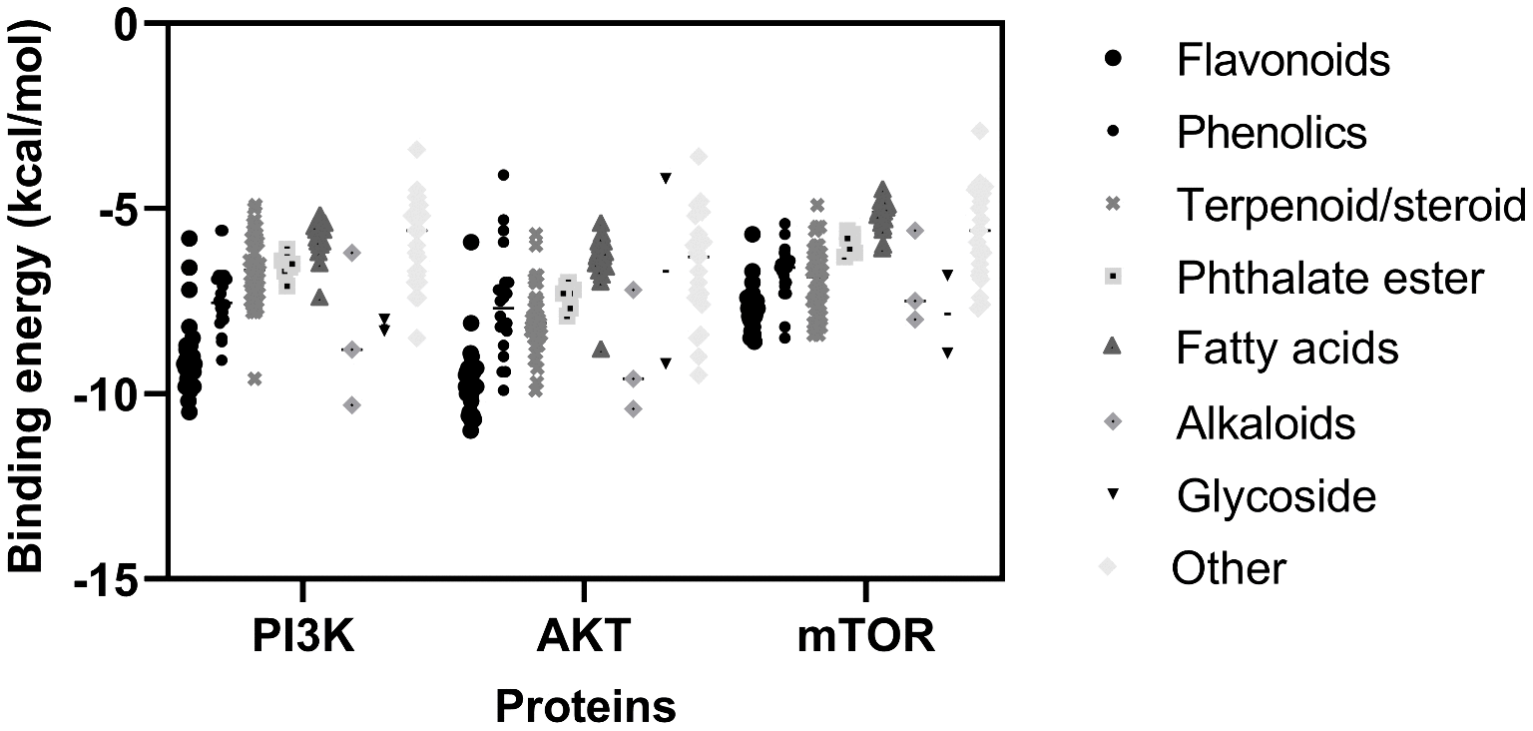
Binding energy of bioactive compounds from *C. amuricus* to receptor proteins.

Building on the re-docking findings, 159 Cyperaceae-derived compounds-previously reported to exhibit anticancer activity-were retrieved from PubMed databases and screened for their binding affinities to receptor proteins, including PI3K (7K6M), AKT (5KCV), and mTOR (4JSV) (S3 Table). Molecular docking simulations were conducted using parameters and protocols validated through the re-docking experiments. The binding energies of these compounds ranged from −10.50 to −2.9 kcal/mol (Fig 7).

As for PI3K, fifteen compounds exhibited strong affinity, with binding energies lower than −9.30 kcal/mol (S4 Table), which is the reference value corresponding to co-crystallized ligand VXY (Table 2). In addition, three compounds (32, 50, 51) displayed binding energies comparable to positive control Ly294002 (−10.20 kcal/mol, S1 Fig). Among these, luteolin 7-O-β-D-glucuronopyranoside-6″-methyl ester (compound 51), a flavonoid, exhibited the strongest interaction, with the lowest binding energy of −10.5 kcal/mol (Table 3).

**Table 3:** Molecular interactions of *C.* *amuricus*-derived compounds with PI3K, AKT, and mTOR proteins.

Protein	Compounds	Types of interactions	Amino acid residues
PI3K	Luteolin 7-O-β-D-glucuronopyranoside-6″-methyl ester	Hydrogen bond	Arg770, Asp810, Tyr836, Val851, Ser854, Asp933
		Carbon-hydrogen bond (C–H⋯O)	His855
		π-Alkyl hydrophobic	Ile800, Ile932, Val850
		π-σ hydrophobic	Ile848, Met922
		π-sulfur bond	Met922
AKT	Luteolin 7-O-β-D-glucuronopyranoside-6″-methyl ester	Hydrogen bond	Asn54, Gln79, Thr211, **Tyr272**, Arg273
		π-Alkyl hydrophobic	**Leu210**
		π-σ hydrophobic	**Val270**
		π-π stacking	Trp80
AKT	Vitexin	Hydrogen bond	Gln79, Thr81, Thr82, Thr211, Val271
		π-Alkyl hydrophobic	Leu264
		π-σ bond	**Val270**
		π-π stacking	Trp80, Asp292
mTOR	Digitoxin	Hydrogen bond	Ser2165, Lys2187, Thr2245, Ser2342, Asp2357
		Carbon-hydrogen bond (C–H⋯O)	Glu2190
		π-Alkyl hydrophobic	Ala2248

**Fig 8:**
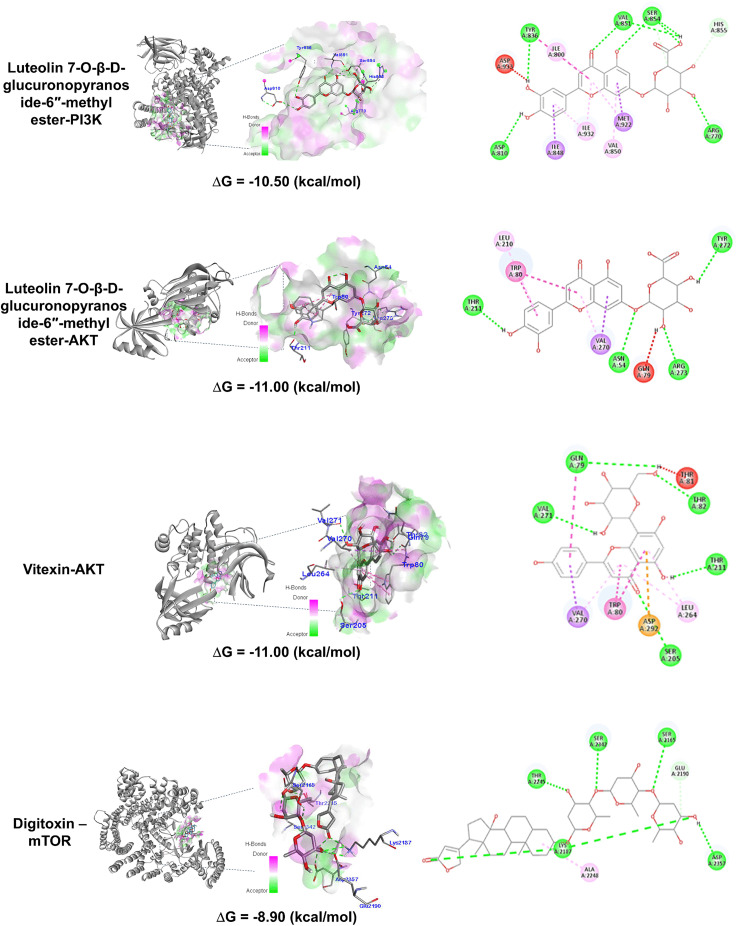
Molecular docking of *C. amuricus*-derived compounds with PI3K/AKT/mTOR protein.

Compound 51 formed hydrogen bonds with key residues at the PI3K active site, including Arg770, Asp810, Tyr836, Val851, Ser854, and Asp933, and a carbon-hydrogen bond with His855. It also exhibited hydrophobic interactions with Ile800, Ile932, Val850, Ile848, and Met922 (Fig 8 and Table 3).

The molecular docking simulations also revealed that the binding energies of Cyperaceae-derived compounds with AKT protein ranging from −3.60 to −11.00 kcal/mol (Fig 7). Although none of the investigated compounds surpassed the binding affinity of the co-crystallized ligand ARQ092 (ΔG = −13.9 kcal/mol, Fig 6) or positive control Ly294002 (ΔG = −11.6 kcal/mol, S1 Fig), ten flavonoids showed strong binding affinity (ΔG ≤ −10 kcal/mol) (S5 Table). Among them, compounds 51 and compound 64 (vitexin) exhibited the strongest binding affinities (ΔG = −11.00 kcal/mol). Molecular interactions show that compound 51 interacted with the residues Asn54, Gln79, Trp80, Leu210, Thr211, Val270, Tyr272, Arg273. Similarly vitexin interacted with Gln79, Trp80, Thr81, Thr82, Thr211, Leu264, Val270, Val271, Asp292 (Table 3). Regarding mTOR, eleven Cyperaceae-derived compounds—primarily flavonoids and their derivatives—showed strong binding affinity, with ΔG values ranging from −8.90 to −8.20 kcal/mol (S6 Table). These values surpassed that of co-crystallized ligand (ΔG = −7 kcal/mol, Fig 6) and were comparable to the positive control Ly294002 (ΔG = −8.50 kcal/mol, S1 Fig). Digitoxin (66) showed the strongest interactions, recording the lowest binding energy (−8.90 kcal/mol). Digitoxin, a cardiac glycoside commonly used to treat heart failure and arrhythmias, demonstrated strong affinity for the highly conserved ATP-binding pocket of mTOR, including residues Ile2163, Ser2165, Lys2187, Glu2190, Tyr2225, Trp2239, Val2240, Thr2245, Ser2342, Asn2343, Met2345, Ile2356, and Asp2357 [36]. Molecular simulations revealed that digitoxin established hydrogen bonds with Ser2165, Lys2187, Thr2245, Ser2342, and Asp2357, and a non-classical C–H⋯O hydrogen bond with Glu2190. Additionally, it engages in hydrophobic interactions with Ala2248 (Table 3).

## Discussion

Hepatocellular carcinoma (HCC), the predominant form of primary liver cancer, remains a leading cause of cancer-related mortality worldwide. Current therapeutic approaches are limited by drug resistance, severe side effects, and high treatment cost, suggesting the urgent need to explore natural compounds as alternative or adjunct therapies. This study combines both *in vitro* evaluation and molecular docking analysis to assess the inhibitory effects of the *C. amuricus* fractionated extracts on HepG2 cells. The EA extract selectively inhibited HCC cell proliferation, which may be attributed to its high phenolic and flavonoid content. Apoptotic induction was confirmed by characteristic nuclear changes. Western blot analysis revealed dose-dependent suppression of Akt (Ser473) and p70S6K (Ser371) phosphorylation, indicating inhibition of the PI3K/AKT/mTOR pathway. Furthermore, molecular docking analysis revealed strong binding affinities between Cyperaceae-derived constituents and key oncogenic targets (PI3K, AKT, mTOR), suggesting that the inhibition of this signaling axis may underlie the anticancer activity of *C. amuricus.*

The EA fraction of *C. amuricus* exhibited selective anti-proliferative activity against HCC. Among the tested extracts, the EA fraction showed the strongest inhibition of HepG2 cell growth (Fig 2), displaying an IC_50_ value of 159.76 µg/mL (Fig 3A). However, it showed no inhibitory effects on HDFa cells, even at high investigated concentrations (150–200 µg/mL) treatment, highlighting its selective cytotoxicity. While this study focused on HepG2 cells, our previous research showed that *C. amuricus* extracts also exert cytotoxic effects on Hep3B cells by inducing G0/G1 cell cycle arrest and activating the mitochondrial-dependent apoptosis pathway [26,37], supproting its the broader anticancer potential of *C. amuricus* across HCC cell lines. The pronounced anticancer activity of the EA fraction may be attributed to its high TPC and TFC (Fig 1), consistent with the well-documented anticancer properties of polyphenols and flavonoids. Flavonoids are known to inhibit the PI3K/Akt/mTOR cascade, leading to cell cycle arrest, apoptosis induction, and suppression of angiogenesis. For examples, epicatechin and epigallocatechin gallate (EGCG) regulate pathways such as NF-κB, MAPK, and PI3K, promoting apoptosis in cancer cells [19]; EGCG also downregulates Akt/mTOR signaling in gastric carcinoma [18]. Collectively, these findings may implicate the flavonoid-mediated pathway modulation in the anticancer efficacy of EA fraction. However, the chemical characterization of its active constituents remains incomplete, limiting mechanistic interpretation and translational potential. Further phytochemical profiling and compound isolation are needed to clarify the molecular drivers of its anticancer efficacy.

Apoptosis induced by EA extract of *C. amuricus* in HepG2 cells was confirmed through characteristic morphological changes and DNA fragmentation. Morphological features of apoptosis, including cell shrinkage, chromatin condensation (pyknosis), nuclear fragmentation (karyorrhexis), and membrane blebbing, were observed following treatment, while HDFa cells maintained normal morphology under the same conditions (Figs 3B and 4A). NAF provided quantitative validation of nuclear changes in apoptotic cells. The significant reduction in NAF at 150–200 µg/mL treatment indicated nuclear shrinkage and structural loss associated with apoptosis (Fig 4B). These findings are consistent with previous studies, displaying a marked and significant reduction in NAF among apoptotic cells compared with controls (P < 0.01), which is interpreted as an indicator of DNA degradation [38]. DNA fragmentation was further confirmed by gel electrophoresis (Fig 4C). DNA fragmentation is mediated by the DNA fragmentation factor (DFF) complex, composed of DFF40 and DFF45. Caspase-dependent cleavage of DFF45 releases DFF40, enabling its dimerization and cleaves DNA at internucleosomal linkers. This process produces fragments of approximately 180 base pairs and their multiples, generating the characteristic “DNA ladder” pattern—an established marker of apoptosis [39].

*C. amuricus* EA extract led to a suppression of phosphorylated Akt (Ser473) and p70S6 Kinase (Ser371) in HepG2 cells (Fig 5), further revealing the molecular mechanisms of its cytotoxicity effects. The phosphorylation of Akt at Ser473—mediated by the mTORC2 complex—is essential for full enzymatic activation, enhancing AKT activity by approximately tenfold following initial phosphorylation at Thr308 by PDK1 [40,41]. The reduction in Ser473 phosphorylation suggests that EA extract disrupts mTORC2-mediated Akt activation, thereby impairing anti-apoptotic signaling. Similarly, phosphorylation of p70S6 at Ser371—regulated by mTORC1—is critical for cap-dependent translation and cell growth [33,42]. The EA extract’s suppression of p70S6 phosphorylation indicates inhibition of mTORC1-mediated translational control, further contributing to its anti-proliferative effects. Together, these findings suggest that EA extract disrupts mTOR-mediated signaling, induces apoptotic effects—evidenced by morphological alterations, NAF reduction, and DNA fragmentation—, supporting its potential as a selective therapeutic agent for HCC.

We based our docking study on compounds reported in Cyperaceae plants, but the actual active components in our extract have not yet been confirmed. Molecular docking analysis showed that the phytoconstituents from the Cyperaceae family, particularly flavonoid compounds, exhibited strong affinity toward key components of the PI3K/AKT/mTOR pathways. PI3Kα comprises two subunits: the catalytic p110α and the regulatory p85α. The p110α subunit contains multiple functional domains, including an N-terminal adaptor binding domain (ABD), a Ras-binding domain, a C2 domain, a helical domain, and a catalytic kinase domain. The ATP-binding site resides between the two lobes of the catalytic domain, separated by a hinge region [8]. This pocket contains highly conserved residues across PI3K isoforms, including Met772, Ser774, Trp780, Pro778, Ile800, Lys802, Tyr836, Ile848, Val850, Val851, Met922, Phe930, Ile932, Asp933 [43]. Notably, both VXY-PI3K and compound 51-PI3K complexes formed similar interactions, including hydrogen bonds with Asp810, Val851, and Asp933, and hydrophobic interactions with Ile848, Val850, and Ile932 (Tables 2 and 3). The positive control Ly294002 also interacted with similar residues like Ile800, Val850, Ile848 and Met922 (S7 Table), highliting the important roles of these residues in ligand binding. These findings are consistent with previous molecular docking studies targeting PI3K. Quinazoline derivatives with nanomolar activities against PI3Kα have been shown to interact with key residues such as Lys802, Asp810, and Val851 [44]. Additional studies emphasized the critical role of hydrogen bonds with Val851 in ligand binding [13], while interaction with Ser854 may contribute to isoform selectivity [12]. In our analysis, the C-H group in the imidazole side chains of His855 acted as a proton donor, forming a non-classical hydrogen bond (NCHB) with an oxygen atom of the 51 compound. Although NCHBs are generally weaker (∆G = −0.5 to −3.7 kcal/mol) than classical hydrogen bonds (∆G = −3.1 to −6.9 kcal/mol), they significantly contribute to ligand binding affinity, selectivity, and conformational stabilization [45,46]. Additionally, compound 51 formed a distinctive hydrophobic interaction through a π-sulfur bond between Met922 and its aromatic ring. Met-aromatic motifs have been reported to provide an additional 1–1.5 kcal/mol of stabilization, playing an important role in high-affinity ligand binding and receptor function [47].

AKT, also known as protein kinase B (PKB), is a serine/threonine-specific kinase belonging to the AGC family. All AKT isoforms share a common structural organization comprising three domains: a pleckstrin homology (PH) domain at the N-terminus, a catalytic kinase domain with ATP-binding site, and a hydrophobic motif (HM) at the C-terminus [48]. PI3K activation induces PIP3 accumulation, promoting AKT recruitment to the cell membrane via PIP3-PH domain interactions. This triggers phosphorylation at regulatory sites, initiating downstream signaling cascades that promote cell proliferation, cell cycle progression, survival, and apoptosis inhibition [7]. Dysregulation of the AKT signal is detected in the development of numerous diseases, including cancer [10], making AKT inhibitors promising candidate for anticancer therapy. AKT inhibitors are primarily classified into two categories: ATP-competitive and allosteric inhibitors. ATP-competitive inhibitors bind the catalytic domain and inhibit all three AKT isoforms simultaneously, whereas allosteric inhibitors target an a pocket formed between the PH and kinase domains, blocking the AKT phosphorylation and activation [7]. Examples of allosteric inhibitors include MK-2206 [15] and ARQ092 [14], which offer improved isoform selectivity. Compounds 51 and 64 exhibit binding interactions similar to co-crystallized ARQ092, forming hydrogen bonds with Thr211 and hydrophobic interactions with Trp80 and Val270 (Tables 2–3). These findings suggest that these residues play a critical role in ligand binding to the allosteric pocket of AKT. Lapierre Jean-Marc et al. reported that hydrogen bonds with Tyr272 and Asp274 help orient drugs to the hydrophobic residues within the PH domain [14]. Trp80 also contributes significantly by forming π-π stacking interactions between its indole group and the aromatic moieties of compounds 51 and 64. These findings are consistent with previous studie. Green et al. reported that an AKT inhibitor mediates AKT inhibition through a solvent-exposed Trp80 residue [41], while computational study of MK-2206 identified Trp80 as a mediator residue of hydrophobic interaction, ligand binding, and drug incorporation into the of AKT1 binding site [15]. Additionally, vitexin formed hydrogen bonds with AMP-activated protein kinase, modulating the downstream AKT/GSK-3β/Nrf2 pathway and attenuating of autoimmune hepatitis [49]. Compound 51—a derivative of luteolin, a flavonoid with documented anticancer activity against breast, colon, prostate, and glioblastoma cancers—further supports the therapeutic potentical of luteolin and its derivatives [50].

The mammalian target of rapamycin (mTOR) is a Ser/Thr kinase with a highly conserved structure, classified into two complexes: mTORC1 and mTORC2. As a central regulator of protein synthesis, mTOR links nutrient sensing to cell growth and is frequently implicated in cancer. Activation of mTORC1 signaling via PI3K and AKT results in the phosphorylation of ribosomal protein S6 kinase 1 (p70S6K) and eukaryotic translation initiation factor 4E-binding protein-1 (4E-BP1) [6]. Digitoxin (compound 66) formed hydrogen bonds and C–H⋯O interactions with residues in the ATP-binding pocket of mTOR (Fig 8). The hydrogen bond with Ser2165 was identified as an important interaction for ligand binding [17], while the π-Alkyl hydrophobic interaction with Ala2248 may enhance complex stability. Digitoxin is commonly associated with Digitalis plants, but interestingly, GC-MS analysis of Cyperus alternifolius detected digitoxin (or an isomeric glycoside) among its phytochemicals [24]. The previous study demonstrated that digitoxin induces apoptosis and inhibits proliferation and migration of colon cancer cells by reducing phosphorylation of mTOR substrates p70S6K and 4E-BP1 [51]. Collectively, these findings support the therapeutic potential of *C. amuricus* as a targeted agent for hepatocellular carcinoma (HCC) via modulation of the PI3K/AKT/mTOR signaling axis.

Despite the promising *in vitro* findings and strong molecular docking correlations, several limitations should be acknowledged. First, the absence of *in vivo* validation restricts the translational relevance of the observed cytotoxic and apoptotic effects. Without animal model data, it remains uncertain whether the identified compounds would exhibit similar efficacy or safety profiles in a physiological context. Future *in vivo* studies will be required to examine the bioavailability of these phytochemicals and to assess their possible systemic toxicity, given the known pharmacological properties of compounds such as digitoxin. Second, the chemical characterization of the active constituents within the ethyl acetate fraction remains incomplete. While docking suggests potential bioactive flavonoids, definitive structural identification and quantification are necessary to confirm their roles. Additionally, factors such as compound bioavailability, metabolic stability, and potential off-target effects warrant further investigation before clinical applicability can be considered.

## Conclusion

This study investigates the *in vitro* antitumor activity of *C. amuricus* fractionated extracts against hepatocellular carcinoma cells, coupled with molecular docking analysis targeting key components of the PI3K/AKT/mTOR signaling axis. The ethyl acetate (EA) fraction showed selective cytotoxicity toward HepG2 cells through apoptosis induction and suppression of the PI3K/AKT/mTOR pathway. Molecular docking revealed that key chemicals compounds from Cyperacea plants exhibit strong interactions with oncogenic targets, suggesting a mechanistic basis for the observed cytotoxicity. Notably, compounds such as luteolin derivatives, vitexin, and digitoxin showed binding affinities comparable to co-crystallized reference ligands. These findings support the therapeutic potential of *C. amuricus* and underscore the need for further i*n vivo* validation and phytochemical characterization to advance its development as a natural anticancer source.

## Supporting information

S1 Fig Molecular docking of Ly294002 with PI3K/AKT/mTOR protein. (TIF)

S1 Table Extraction yield and moisture content of crude and fractions from *C. amuricus.* (DOCX)

S2 Table Redocking of FDA-approved drugs to target receptor proteins. (DOCX)

S3 Table Molecular docking screening of *C.* *amuricus*-derived compounds targeting PI3K, AKT, and mTOR. (DOCX)

S4 Table High binding affinity of *C.* *amuricus*-derived compounds toward PI3K. (DOCX)

S5 Table High binding affinity of *C.* *amuricus*-derived compounds toward AKT. (DOCX)

S6 Table High binding affinity of *C.* *amuricus*-derived compounds toward mTOR. (DOCX)

S7 Table Molecular interactions of Ly294002 with PI3K, AKT, and mTOR proteins. (DOCX)

S1 File Raw images. (TIF)

S2 File Grpahical abstract. (TIF)

## Abbreviations

AKT: protein kinase B
PI3K: Phosphoinositide 3-kinase
mTOR: mammalian target of rapamycin
RMSD: root-mean-square deviation
HCC: Hepatocellular carcinoma
NCHB: non-classical hydrogen bond.

## References

[1] Ferlay JEM, Lam F, Laversanne M, Colombet M, Mery L, Piñeros M, et al. Global cancer observatory: cancer today. Lyon, France: International Agency for Research on Cancer; 2024. https://gco.iarc.who.int/today

[2] IARC. Predictions of the future cancer incidence and mortality burden worldwide up until 2050. IARC – International Agency for Research on Cancer; 2024.

[3] Ferenci P, Fried M, Labrecque D, Bruix J, Sherman M, Omata M, et al. Hepatocellular carcinoma (HCC). J Clin Gastroenterol. 2010;44(4):239–45. doi: 10.1097/mcg.0b013e3181d46ef2 20216082

[4] Rahbari NN, Mehrabi A, Mollberg NM, Müller SA, Koch M, Büchler MW, et al. Hepatocellular Carcinoma. Annal Surg. 2011;253(3):453–69. doi: 10.1097/sla.0b013e31820d944f 21263310

[5] Alawyia B, Constantinou C. Hepatocellular carcinoma: a narrative review on current knowledge and future prospects. Curr Treat Options Oncol. 2023;24(7):711–24. doi: 10.1007/s11864-023-01098-9 37103744

[6] Tian L-Y, Smit DJ, Jücker M. The Role of PI3K/AKT/mTOR Signaling in Hepatocellular Carcinoma Metabolism. Int J Mol Sci. 2023;24(3):2652. doi: 10.3390/ijms24032652 36768977 PMC9916527

[7] Jiang N, Dai Q, Su X, Fu J, Feng X, Peng J. Role of PI3K/AKT pathway in cancer: the framework of malignant behavior. Mol Biol Rep. 2020;47(6):4587–629. doi: 10.1007/s11033-020-05435-1 32333246 PMC7295848

[8] Zhang M, Jang H, Nussinov R. PI3K inhibitors: review and new strategies. Chem Sci. 2020;11(23):5855–65. doi: 10.1039/d0sc01676d 32953006 PMC7472334

[9] Sun EJ, Wankell M, Palamuthusingam P, McFarlane C, Hebbard L. Targeting the PI3K/Akt/mTOR pathway in hepatocellular carcinoma. Biomedicines. 2021;9(11):1639. doi: 10.3390/biomedicines9111639 34829868 PMC8615614

[10] Vasudevan KM, Barbie DA, Davies MA, Rabinovsky R, McNear CJ, Kim JJ, et al. AKT-independent signaling downstream of oncogenic PIK3CA mutations in human cancer. Cancer Cell. 2009;16(1):21–32. doi: 10.1016/j.ccr.2009.04.012 19573809 PMC2752826

[11] Ul Bari W, Zahoor M, Zeb A, Sahibzada MUK, Ullah R, Shahat AA, et al. Isolation, pharmacological evaluation and molecular docking studies of bioactive compounds from Grewia optiva. Drug Des Devel Ther. 2019;13:3029–36. doi: 10.2147/DDDT.S220510 31692531 PMC6717395

[12] Sabbah DA, Vennerstrom JL, Zhong H. Docking studies on isoform-specific inhibition of phosphoinositide-3-kinases. J Chem Inf Model. 2010;50(10):1887–98. doi: 10.1021/ci1002679 20866085 PMC4480772

[13] Al Hasan M, Sabirianov M, Redwine G, Goettsch K, Yang SX, Zhong HA. Binding and selectivity studies of phosphatidylinositol 3-kinase (PI3K) inhibitors. J Mol Graph Model. 2023;121:108433. doi: 10.1016/j.jmgm.2023.108433 36812742

[14] Lapierre J-M, Eathiraj S, Vensel D, Liu Y, Bull CO, Cornell-Kennon S, et al. Discovery of 3-(3-(4-(1-Aminocyclobutyl)phenyl)-5-phenyl-3H-imidazo[4,5-b]pyridin-2-yl)pyridin-2-amine (ARQ 092): an orally bioavailable, selective, and potent allosteric AKT inhibitor. J Med Chem. 2016;59(13):6455–69. doi: 10.1021/acs.jmedchem.6b00619 27305487

[15] Rehan M, Beg MA, Parveen S, Damanhouri GA, Zaher GF. Computational insights into the inhibitory mechanism of human AKT1 by an orally active inhibitor, MK-2206. PLoS ONE. 2014;9(10):e109705. doi: 10.1371/journal.pone.0109705 PMC4201482 25329478

[16] Wang L, Chen L, Yu M, Xu L-H, Cheng B, Lin Y-S, et al. Discovering new mTOR inhibitors for cancer treatment through virtual screening methods and in vitro assays. Sci Rep. 2016;6(1). doi: 10.1038/srep18987 PMC4702177 26732172

[17] Oluić J, Nikolic K, Vucicevic J, Gagic Z, Filipic S, Agbaba D. 3D-QSAR, virtual screening, docking and design of dual PI3K/mTOR inhibitors with enhanced antiproliferative activity. Comb Chem High Throughput Screen. 2017;20(4):292–303. doi: 10.2174/1386207320666170427143858 28460621

[18] Zughaibi TA, Suhail M, Tarique M, Tabrez S. Targeting PI3K/Akt/mTOR pathway by different flavonoids: a cancer chemopreventive approach. Int J Mol Sci. 2021;22(22):12455. doi: 10.3390/ijms222212455 34830339 PMC8621356

[19] Abotaleb M, Samuel SM, Varghese E, Varghese S, Kubatka P, Liskova A, et al. Flavonoids in cancer and apoptosis. Cancers (Basel). 2018;11(1):28. doi: 10.3390/cancers11010028 30597838 PMC6357032

[20] Taheri Y, Herrera-Bravo J, Huala L, Salazar LA, Sharifi-Rad J, Akram M, et al. *Cyperus* spp.: a review on phytochemical composition, biological activity, and health-promoting effects. Oxid Med Cell Longev. 2021;2021:4014867. doi: 10.1155/2021/4014867 34539969 PMC8443348

[21] Lee S, Choi H, Jeon H, Baek NI, Kim SH, Kim HJ, et al. Antioxidant phenolic components from the whole plant extract of *Cyperus amuricus* Max. Korean J Pharm. 2008;39(3):233–6.

[22] Sharma N, Sharma VK, Seo S-Y. Screening of some medicinal plants for anti-lipase activity. J Ethnopharmacol. 2005;97(3):453–6. doi: 10.1016/j.jep.2004.11.009 15740880

[23] Rocha FG, Brandenburg M de M, Pawloski PL, Soley B da S, Costa SCA, Meinerz CC, et al. Preclinical study of the topical anti-inflammatory activity of Cyperus rotundus L. extract (Cyperaceae) in models of skin inflammation. J Ethnopharmacol. 2020;254:112709. doi: 10.1016/j.jep.2020.112709 32109543

[24] Al-Gara a, Neepal I, Abu-Serag NA, Alee SKA, Bahadly ZKA. Analysis of bioactive phytochemical compound of (Cyperus alternifolius L.) by using gas chromatography–mass spectrometry. In: I OP conference series: materials science and engineering. IOP Publishing; 2019.

[25] Bezerra JJL, Pinheiro AAV, de Oliveira AFM. Chemical composition and anticancer activity of essential oils from cyperaceae species: a comprehensive review. Sci Pharm. 2025;93(1):9. doi: 10.3390/scipharm93010009

[26] Pham HHT, Seong Y-A, Ngabire D, Oh C-W, Kim G-D. Cyperus amuricus induces G1 arrest and apoptosis through endoplasmic reticulum stress and mitochondrial signaling in human hepatocellular carcinoma Hep3B cells. J Ethnopharmacol. 2017;208:157–64. doi: 10.1016/j.jep.2017.07.002 28684299

[27] Kupina S, Fields C, Roman MC, Brunelle SL. Determination of total phenolic content using the Folin-C assay: single-laboratory validation, first action 2017.13. J AOAC Int. 2018;101(5):1466–72. doi: 10.5740/jaoacint.18-0031 29895350

[28] Martono Y, Yanuarsih F, Aminu N, Muninggar J. Fractionation and determination of phenolic and flavonoid compound from Moringa oleifera leaves. In: Journal of Physics: Conference Series. IOP Publishing; 2019.

[29] Cragg GM, Pezzuto JM. Natural products as a vital source for the discovery of cancer chemotherapeutic and chemopreventive agents. Med Princ Pract. 2015;25(Suppl. 2):41–59. doi: 10.1159/000443404 26679767 PMC5588531

[30] Wang K, Fan H, Chen Q, Ma G, Zhu M, Zhang X, et al. Curcumin inhibits aerobic glycolysis and induces mitochondrial-mediated apoptosis through hexokinase II in human colorectal cancer cells in vitro. Anticancer Drugs. 2015;26(1):15–24. doi: 10.1097/CAD.0000000000000132 25229889

[31] Eidet JR, Pasovic L, Maria R, Jackson CJ, Utheim TP. Objective assessment of changes in nuclear morphology and cell distribution following induction of apoptosis. Diagn Pathol. 2014;9:92. doi: 10.1186/1746-1596-9-92 24885713 PMC4048047

[32] Nguyen L-TT, Thi Le N-H, Thi Ta HK, Dang Nguyen K. Isolation of DNA from Arthrospira platensis and whole blood using magnetic nanoparticles (Fe3O4@OA and Fe3O4@OA@SiO2). J Anal Sci Technol. 2022;13(1). doi: 10.1186/s40543-022-00337-2

[33] Yang H, Rudge DG, Koos JD, Vaidialingam B, Yang HJ, Pavletich NP. mTOR kinase structure, mechanism and regulation. Nature. 2013;497(7448):217–23. doi: 10.1038/nature12122 23636326 PMC4512754

[34] Häcker G. The morphology of apoptosis. Cell Tissue Res. 2000;301(1):5–17. doi: 10.1007/s004410000193 10928277

[35] Pietruś W, Kafel R, Bojarski AJ, Kurczab R. Hydrogen bonds with fluorine in ligand-protein complexes-the PDB analysis and energy calculations. Molecules. 2022;27(3):1005. doi: 10.3390/molecules27031005 35164270 PMC8838457

[36] Tanneeru K, Guruprasad L. Ligand-based 3-D pharmacophore generation and molecular docking of mTOR kinase inhibitors. J Mol Model. 2012;18(4):1611–24. doi: 10.1007/s00894-011-1184-3 21805127

[37] Pham HHT, Seong Y-A, Oh C-W, Kim G-D. The herbal medicine Cyperus amuricus inhibits proliferation of human hepatocellular carcinoma Hep3B cells by inducing apoptosis and arrest at the G0/G1 cell cycle phase. Int J Oncol. 2016;49(5):2046–54. doi: 10.3892/ijo.2016.3698 27667556

[38] Helmy IM, Azim AMA. Efficacy of ImageJ in the assessment of apoptosis. Diagn Pathol. 2012;7:15. doi: 10.1186/1746-1596-7-15 22309648 PMC3307432

[39] Majtnerová P, Roušar T. An overview of apoptosis assays detecting DNA fragmentation. Mol Biol Rep. 2018;45(5):1469–78. doi: 10.1007/s11033-018-4258-9 30022463

[40] Alessi DR, Caudwell FB, Andjelkovic M, Hemmings BA, Cohen P. Molecular basis for the substrate specificity of protein kinase B; comparison with MAPKAP kinase-1 and p70 S6 kinase. FEBS Lett. 1996;399(3):333–8. doi: 10.1016/s0014-5793(96)01370-1 8985174

[41] Green CJ, Göransson O, Kular GS, Leslie NR, Gray A, Alessi DR, et al. Use of Akt inhibitor and a drug-resistant mutant validates a critical role for protein kinase B/Akt in the insulin-dependent regulation of glucose and system A amino acid uptake. J Biol Chem. 2008;283(41):27653–67. doi: 10.1074/jbc.M802623200 18669636

[42] Hay N, Sonenberg N. Upstream and downstream of mTOR. Genes Dev. 2004;18(16):1926–45. doi: 10.1101/gad.1212704 15314020

[43] Tantawy AH, El-Behairy MF, Abd-Allah WH, Jiang H, Wang M-Q, Marzouk AA. Design, synthesis, biological evaluation, and computational studies of novel fluorinated candidates as PI3K inhibitors: targeting fluorophilic binding sites. J Med Chem. 2021;64(23):17468–85. doi: 10.1021/acs.jmedchem.1c01674 34791873

[44] Al-Ashmawy AAK, Elokely KM, Perez-Leal O, Rico M, Gordon J, Mateo G, et al. Discovery and SAR of novel disubstituted quinazolines as dual PI3Kalpha/mTOR inhibitors targeting breast cancer. ACS Med Chem Lett. 2020;11(11):2156–64. doi: 10.1021/acsmedchemlett.0c00289 33214824 PMC7667832

[45] Horowitz S, Trievel RC. Carbon-oxygen hydrogen bonding in biological structure and function. J Biol Chem. 2012;287(50):41576–82. doi: 10.1074/jbc.R112.418574 23048026 PMC3516709

[46] Johnston RC, Cheong PH-Y. C-H···O non-classical hydrogen bonding in the stereomechanics of organic transformations: theory and recognition. Org Biomol Chem. 2013;11(31):5057–64. doi: 10.1039/c3ob40828k 23824256

[47] Valley CC, Cembran A, Perlmutter JD, Lewis AK, Labello NP, Gao J, et al. The methionine-aromatic motif plays a unique role in stabilizing protein structure. J Biol Chem. 2012;287(42):34979–91. doi: 10.1074/jbc.M112.374504 22859300 PMC3471747

[48] Halder AK, Cordeiro MNDS. AKT inhibitors: the road ahead to computational modeling-guided discovery. Int J Mol Sci. 2021;22(8):3944. doi: 10.3390/ijms22083944 33920446 PMC8070654

[49] Zhang L, Chen D, Tu Y, Sang T, Pan T, Lin H, et al. Vitexin attenuates autoimmune hepatitis in mouse induced by syngeneic liver cytosolic proteins via activation of AMPK/AKT/GSK-3β/Nrf2 pathway. Eur J Pharmacol. 2022;917:174720. doi: 10.1016/j.ejphar.2021.174720 34953801

[50] Imran M, Rauf A, Abu-Izneid T, Nadeem M, Shariati MA, Khan IA, et al. Luteolin, a flavonoid, as an anticancer agent: a review. Biomed Pharmacother. 2019;112:108612. doi: 10.1016/j.biopha.2019.108612 30798142

[51] Mi C, Cao X, Ma K, Wei M, Xu W, Lin Y, et al. Digitoxin promotes apoptosis and inhibits proliferation and migration by reducing HIF-1α and STAT3 in KRAS mutant human colon cancer cells. Chem Biol Interact. 2022;351:109729. doi: 10.1016/j.cbi.2021.109729 34717917

