# Supplementary material for: Bioactivity of Cyperus amuricus extracts against hepatocellular carcinoma and molecular docking analysis targeting the PI3K/AKT/mTOR pathway
Source: PLoS One. 2026 Jan 9;21(1):e0340868. doi: 10.1371/journal.pone.0340868 (PMC12788648; doi:10.1371/journal.pone.0340868)
Supplement: S1 Table — (DOCX) [file pone.0340868.s002.docx]

Bioactivity of *Cyperus amuricus* Extracts Against Hepatocellular Carcinoma and Molecular Docking Analysis Targeting the PI3K/AKT/mTOR Pathway

**Thanh Luan Nguyen^1^, Thanh Khoi Tu^2,3^, Thien-Vy Phan^4^, Chanh M. Nguyen^5,6^ Khoa D. Nguyen^5,6^ Minh Quan Pham^7,8^,** **Hai Ha Pham Thi^2,3*^**

^1^ HUTECH Institute of Applied Science, HUTECH University, Ho Chi Minh City, Viet Nam

^2^ Center for Hi-Tech Development, Nguyen Tat Thanh University, Saigon Hi-Tech Park, Ho Chi Minh City, Vietnam.

^3^ NTT Hi-Tech Institute, Nguyen Tat Thanh University, Ho Chi Minh City, Vietnam.

^4^ Faculty of Pharmacy, Nguyen Tat Thanh University, Ho Chi Minh City, Vietnam

^5^ Institute of Applied Science and Technology, Van Lang School of Technology, Van Lang University, Ho Chi Minh City, Vietnam

^6^ Faculty of Applied Technology, Van Lang School of Technology, Van Lang University, Ho Chi Minh City, Vietnam

^7^ Institute of Natural Products Chemistry, Vietnam Academy of Science and Technology, Hanoi, Vietnam.

^8^ Graduate University of Science and Technology, Vietnam Academy of Science and Technology (VAST), Hanoi, Vietnam.

***** **Corresponding author:**

Email: [pthha@ntt.edu.vn](mailto:pthha@ntt.edu.vn) (Ph.D.)

**Short Title**

*Cyperus amuricus:* Anti-Hepatocellular Carcinoma and Molecular Docking Targeting the PI3K/AKT/mTOR Pathway

## Supporting information

**S1 Table. Extraction yield and moisture content of crude and fractions from *Cyperus amuricus.*** Abbreviations: GAE (gallic acid equivalent), QE (quercetin equivalent), and extraction solvents: Methanol (MeOH), Hexane (Hex), Chloroform (TCM), and Ethyl Acetate (EA).

| **Extracts** | **Weight** | **Yield** | **Moisture content** |
| --- | --- | --- | --- |
|  | **(gram)** | **(%)** | **(%)** |
| MeOH | 52.64 ± 5.42 | 17.55 ± 1.81 | 14.67 |
| Hex | 4.42 ± 0.89 | 8.37 ± 1.33 | 13.36 |
| TCM | 1.17 ± 0.28 | 2.20 ± 0.30 | 9.31 |
| EA | 2.23 ± 0.84 | 4.24 ± 1.02 | 9.17 |
| Water | 40.02 ± 0.84 | 76.37 ± 5.60 | 17.21 |
