# Supplementary material for: Bioactivity of Cyperus amuricus extracts against hepatocellular carcinoma and molecular docking analysis targeting the PI3K/AKT/mTOR pathway
Source: PLoS One. 2026 Jan 9;21(1):e0340868. doi: 10.1371/journal.pone.0340868 (PMC12788648; doi:10.1371/journal.pone.0340868)
Supplement: S2 Table — (DOCX) [file pone.0340868.s003.docx]

Bioactivity of *Cyperus amuricus* Extracts Against Hepatocellular Carcinoma and Molecular Docking Analysis Targeting the PI3K/AKT/mTOR Pathway

**Thanh Luan Nguyen^1^, Thanh Khoi Tu^2,3^, Thien-Vy Phan^4^, Chanh M. Nguyen^5,6^ Khoa D. Nguyen^5,6^ Minh Quan Pham^7,8^,** **Hai Ha Pham Thi^2,3*^**

^1^ HUTECH Institute of Applied Science, HUTECH University, Ho Chi Minh City, Viet Nam

^2^ Center for Hi-Tech Development, Nguyen Tat Thanh University, Saigon Hi-Tech Park, Ho Chi Minh City, Vietnam.

^3^ NTT Hi-Tech Institute, Nguyen Tat Thanh University, Ho Chi Minh City, Vietnam.

^4^ Faculty of Pharmacy, Nguyen Tat Thanh University, Ho Chi Minh City, Vietnam

^5^ Institute of Applied Science and Technology, Van Lang School of Technology, Van Lang University, Ho Chi Minh City, Vietnam

^6^ Faculty of Applied Technology, Van Lang School of Technology, Van Lang University, Ho Chi Minh City, Vietnam

^7^ Institute of Natural Products Chemistry, Vietnam Academy of Science and Technology, Hanoi, Vietnam.

^8^ Graduate University of Science and Technology, Vietnam Academy of Science and Technology (VAST), Hanoi, Vietnam.

***** **Corresponding author:**

Email: [pthha@ntt.edu.vn](mailto:pthha@ntt.edu.vn) (Ph.D.)

**Short Title**

*Cyperus amuricus:* Anti-Hepatocellular Carcinoma and Molecular Docking Targeting the PI3K/AKT/mTOR Pathway

## Supporting information

**S2 Table. Redocking of FDA-approved drugs to target receptor proteins**

| **Protein** | **Drug** | **CID** | **∆G** | **RMSD** |
| --- | --- | --- | --- | --- |
|  |  |  | **(kcal/mol)** | **(Å)** |
| PI3K | Alpelisib | 56649450 | -10.0 | 2.901 |
| AKT | Capivasertib | 25227436 | -9.80 | 1.777 |
