# Supplementary material for: Bioactivity of Cyperus amuricus extracts against hepatocellular carcinoma and molecular docking analysis targeting the PI3K/AKT/mTOR pathway
Source: PLoS One. 2026 Jan 9;21(1):e0340868. doi: 10.1371/journal.pone.0340868 (PMC12788648; doi:10.1371/journal.pone.0340868)
Supplement: S3 Table — (DOCX) [file pone.0340868.s004.docx]

Bioactivity of *Cyperus amuricus* Extracts Against Hepatocellular Carcinoma and Molecular Docking Analysis Targeting the PI3K/AKT/mTOR Pathway

**Thanh Luan Nguyen^1^, Thanh Khoi Tu^2,3^, Thien-Vy Phan^4^, Chanh M. Nguyen^5,6^ Khoa D. Nguyen^5,6^ Minh Quan Pham^7,8^,** **Hai Ha Pham Thi^2,3*^**

^1^ HUTECH Institute of Applied Science, HUTECH University, Ho Chi Minh City, Viet Nam

^2^ Center for Hi-Tech Development, Nguyen Tat Thanh University, Saigon Hi-Tech Park, Ho Chi Minh City, Vietnam.

^3^ NTT Hi-Tech Institute, Nguyen Tat Thanh University, Ho Chi Minh City, Vietnam.

^4^ Faculty of Pharmacy, Nguyen Tat Thanh University, Ho Chi Minh City, Vietnam

^5^ Institute of Applied Science and Technology, Van Lang School of Technology, Van Lang University, Ho Chi Minh City, Vietnam

^6^ Faculty of Applied Technology, Van Lang School of Technology, Van Lang University, Ho Chi Minh City, Vietnam

^7^ Institute of Natural Products Chemistry, Vietnam Academy of Science and Technology, Hanoi, Vietnam.

^8^ Graduate University of Science and Technology, Vietnam Academy of Science and Technology (VAST), Hanoi, Vietnam.

***** **Corresponding author:**

Email: [pthha@ntt.edu.vn](mailto:pthha@ntt.edu.vn) (Ph.D.)

**Short Title**

*Cyperus amuricus:* Anti-Hepatocellular Carcinoma and Molecular Docking Targeting the PI3K/AKT/mTOR Pathway

## Supporting information

**S3 Table. Molecular Docking Screening of Cyperus amuricus-Derived Compounds Targeting PI3K, AKT, and mTOR**

|  |  |  |  |  |  |  |
| --- | --- | --- | --- | --- | --- | --- |
| **No.** | **Compound CID** | **Compounds** | **Class** | **PI3K** | **AKT** | **mTOR** |
|  |  |  |  | **7k6m** | **5kcv** | **4jsv** |
| 1 | 12306048 | (-)-alpha-Cadinene | Sesquiterpene | -6.5 | -7.7 | -6.0 |
| 2 | 12310804 | (2S,3S)-2,3-Dihydroxy-2 isopropylbutyric acid [(5R,6S)-6 hydroxy-1-azabicyclo[3.3.0]oct-3 | Alkaloid | -6.2 | -7.2 | -5.6 |
| 3 | 10787 | 1,2,4-Benzenetriol | Benzentriol | -5.1 | -5.2 | -4.6 |
| 4 | 549994 | 1-Bromo-11-iodoundecane | Hydrocarbon | -5.2 | -5.1 | -4.3 |
| 5 | 28813 | 1-Butyl 2-isobutyl phthalate; 2 Methylpropyl butyl phthalate; Butyl isobutyl phthalate | A phthalate ester | -6.4 | -7.1 | -6.3 |
| 6 | 565553 | 2-(Benzyloxymethyl)-5- methylfuran | Furan | -6.7 | -7.0 | -6.1 |
| 7 | 135833395 | 2,7-diphenyl-1,6-dioxopyridazino[4,5,2',3']pyrrolo[4',5'-d]pyridazine | Alkaloid | -10.3 | -10.4 | -8.0 |
| 8 | 15267 | 2-methylpentadecane | Hydrocarbon | -5.6 | -5.7 | -4.8 |
| 9 | 566206 | 2-Pyrazoline, 5-ethyl-1,4-dimethyl | Furan | -4.9 | -4.8 | -4.4 |
| 10 | 163183610 | 3,4,5,4'-Tetramethoxystilbene | Resveratrol | -7.9 | -8.2 | -6.5 |
| 11 | 7121 | 3,4-dimethoxybenzoic acid (Veratric acid) | Benzoic acid | -5.6 | -5.9 | -5.7 |
| 12 | 530546 | 3-cyclopentylpropionic acid, tridecyl ester | Diterpene | -6.2 | -6.8 | -4.9 |
| 13 | 135 | 4-hydroxybenzoic acid | A monohydroxybenzoic acid, | -5.6 | -5.6 | -5.4 |
| 14 | 240147 | 4-Mercaptophenol |  | -4.6 | -4.8 | -4.4 |
| 15 | 537099 | 4-Vinylcholestan-3-ol | Phytosterol | -5.8 | -6.9 | -8.1 |
| 16 | 16242857 | 5,7,3’-trihydroxy-5’-methoxy-8-prenylflavan | Flavonoid | -9.1 | -10.2 | -8.6 |
| 17 | 6482974 | 5,7,8-trimethoxycoumarin | Coumarin | -7.1 | -7.3 | -6.5 |
| 18 | 23403 | 5,7-dimethoxy-8-(gamma,gamma-dimethylallyloxy) coumarin | Coumarin | -7.5 | -8.2 | -7.1 |
| 19 | 2775 | 5,7-dimethoxycoumarin (citropten) | Coumarin | -6.9 | -7.2 | -6.5 |
| 20 | 237332 | 5-Hydroxymethylfurfural | Furan | -4.7 | -4.9 | -4.4 |
| 21 | 42607778 | 6,3',4'-trihydroxy-4-methoxy-7-methylaurone | Flavonoid | -9.1 | -9.8 | -7.9 |
| 22 | 5324625 | 7-(gamma,gamma-dimethylallyloxy)-8-methoxycoumarin | Coumarin | -7.7 | -8.3 | -6.7 |
| 23 | 16242857 | 7,3’-dihydroxy-5,5’-dimethoxy-8-prenylflavan | Flavonoid | -9.1 | -10.6 | -8.4 |
| 24 | 142768 | 7,8-dimethoxycoumarin | Coumarin | -6.8 | -7.2 | -6.1 |
| 25 | 273568 | 7-Isopropenyl-1,4a-dimethyl-4,4a,5,6,7,8-hexahydro-3H-naphthalen-2-one | Sesquiterpenoid | -6.4 | -8.3 | -6.7 |
| 27 | 538453 | 12,15-Octadecadiynoic acid, methyl ester | Fatty acid | -5.9 | -6.6 | -5.3 |
| 28 | 557439 | 13-heptadecyn-1-ol | Fatty alcohol | -5.7 | -5.8 | -5.0 |
| 29 | 5280442 | Acacetin | Flavonoid | -9.4 | -9.3 | -7.9 |
| 30 | 60961 | Adenosine | Ribonucleoside | -6.4 | -7.4 | -5.9 |
| 31 | 6452086 | alpha-Cyperone | Sesquiterpenoids | -7.4 | -8.9 | -7.4 |
| 32 | 9898327 | alpha-D-Glucopyranoside | Trisaccharide | -6.7 | -7.4 | -6.2 |
| 33 | 6654 | alpha-PINENE | Terpene | -5.4 | -5.7 | -6.0 |
| 34 | 10856614 | alpha-selinene | Sesquiterpene hydrocarbon | -7.7 | -8.3 | -7.6 |
| 35 | 11808890 | Anticopalic acid | Diterpene | -7.5 | -9.7 | -7.9 |
| 36 | 5280443 | Apigenin | Flavone | -9.3 | -9.4 | -7.9 |
| 37 | 165536 | Aristolone | Sesquiterpenoid | -5.0 | -7.7 | -6.2 |
| 38 | 91354 | Aromandendrene | Sesquiterpenoid | -7.0 | -8.7 | -7.3 |
| 39 | 10545 | Ascaridole epoxide | Terpenoid | -5.2 | - | - |
| 40 | 5281220 | Aureusidin | Flavonoid | -9.2 | -9.8 | -7.7 |
| 41 | 2347 | Benzyl butyl phthalate | A phthalate ester | -7.1 | -7.9 | -6.2 |
| 42 | 11586487 | beta-himachalene | Sesquiterpenoid | -6.9 | -8.3 | -7.1 |
| 43 | 101596917 | beta-Maaliene | Sesquiterpene | -7.0 | -8.2 | -6.4 |
| 44 | 442393 | beta-selinene | Sesquiterpene hydrocarbon | -7.7 | -8.0 | -7.7 |
| 45 | 222284 | β-sitosterol | Phytosterol | -5.9 | -8.5 | -8.2 |
| 46 | 117301 | Bisabolol oxide II | Oxolanes | -7.0 | -8.4 | -6.7 |
| 47 | 5354342 | Butyl oleate | Fatty acid | -6.0 | -6.2 | -5.4 |
| 48 | 6124299 | Caffeoylmalic Acid | an ester of caffeic acid and malic acid | -7.3 | -7.9 | -6.6 |
| 49 | 5315832 | Caffeoylquinic acid | Quinic acid derivative | -7.8 | -9.4 | -7.3 |
| 50 | 1794427 | Chlorogenic acid | a cinnamate ester/ polyphenolic compounds | -7.6 | -9.4 | -7.0 |
| 51 | 457801 | Clionasterol | Phytosterol | -6.5 | -8.3 | -7.9 |
| 52 | 12303902 | Copaene | Sesquiterpene | -6.0 | -7.5 | -7.2 |
| 53 | 178931 | Corymbolone | Sesquiterpenoid | -6.6 | -8.4 | -6.5 |
| 54 | 323 | Coumarin | Chromenone (benzo-*α*-pyrone) | -6.8 | -7.0 | -6.2 |
| 55 | 160491 | Cyclocolorenone | Ketone group | -7.0 | -8.5 | -6.8 |
| 56 | 533851 | Cyclodododecanol, 1-aminomethyl | Alcohols and polyols, Lactones | -6.3 | -7.7 | -5.6 |
| 57 | 117898 | Cyperaquinone | *p*-quinones and an organic heterotricyclic compound | -8.5 | -9.0 | -7.3 |
| 58 | 12308843 | Cyperene | Sesquiterpene hydrocarbon | -5.3 | -7.0 | -5.5 |
| 59 | 14076601 | Cyperol | Sesquiterpenoid | -6.7 | -8.1 | -6.7 |
| 60 | 12308848 | Cyperotundol | Flavonoid | -7.2 | -8.9 | -7.0 |
| 61 | 118722630 | Cyperotundone | Sesquiterpenoid | -5.5 | -7.4 | -6.1 |
| 62 | 550072 | Dasycarpidan-1-methanol, acetate (ester) | Terpenoid | -7.8 | -9.1 | -7.7 |
| 63 | 3026 | Dibutyl phthalate | A phthalate ester | -6.1 | -7.0 | -5.6 |
| 64 | 91728336 | Diethylmalonic acid, pentyl 3 phenoxybenzyl ester | Fatty acid | -7.4 | -8.8 | -6.0 |
| 65 | 441207 | Digitoxin | Cardiac glycoside | -8.3 | -4.2 | -8.9 |
| 66 | 5281612 | Diosmetin | Flavonoid | -9.8 | -9.3 | -7.7 |
| 67 | 249496 | Di-sec-butyl phthalate | The esterification of isobutanol and phthalic anhydride | -6.6 | -7.2 | -5.8 |
| 68 | 10851 | Dodecanoic acid, 1,2,3-propanetriyl ester | Fatty acid | -5.5 | -5.9 | -5.1 |
| 69 | 68238 | Duroquinones | Benzoquinones | -6.8 | -7.3 | -6.5 |
| 70 | 5281855 | Ellagic acid | Polyphenol | -9.1 | -9.9 | -8.2 |
| 71 |  | Epiorientin | Flavonoid | -9.2 | -10.6 | -8.1 |
| 72 | 13019955 | Ergost-5-en-3-ol, (3beta)-campesterol | Sterol | -6.6 | -7.5 | -8.1 |
| 73 | 54152120 | Ergosta-5,22-dien-3-ol | Sterol | -5.8 | -9.1 | -8.4 |
| 74 | 222285 | Erythritol | Ancol | -3.8 | -4.3 | -3.3 |
| 75 | 228944 | Estra-1,3,5(10)-trien-17β-ol | Steroid | -9.6 | -9.9 | -8.4 |
| 76 | 6452096 | Ethyl iso-allocholate | Trisaccharide | -5.6 | -5.9 | -6.9 |
| 77 | 91694274 | ethyl-alpha-D-glucopyranoside | Glucopyranoside | -4.9 | -6.0 | -4.4 |
| 78 | 5283646 | γ-Ergostenol | Sterol | -6.6 | -9.3 | -8.2 |
| 79 | 577062 | Gamma-Himachalene | Sesquiterpenoid | -7.3 | -8.5 | -7.0 |
| 80 | 159459366 | Glyceryl linolenate | alpha-linolenic acid | -6.2 | -7.0 | -6.1 |
| 81 | 5312413 | Hexadecenoic acid, z-11 | Fatty acid | -6.0 | -6.4 | -5.2 |
| 82 | 5282108 | Irisone (4-(2,6,6-Trimethyl-2-cyclohexen-1-yl)-3-buten-2-one) | Terpene | -6.4 | -7.0 | -6.6 |
| 83 | 10399139 | Isocurcumenol | Sesquiterpenoid | -5.9 | -7.9 | -6.2 |
| 84 | 5281654 | Isorhamnetin | Flavonoid | -9.3 | -9.3 | -7.6 |
| 85 | 162350 | Isovitexin | Apigenin flavone glycoside | -8.7 | -9.9 | -7.6 |
| 86 | 10470863 | Kaempferol 3,5-dimethyl ether | Flavonoid | -8.2 | -9.4 | -7.3 |
| 87 | 5280862 | Kaempferol 3-methyl ether | Flavonoid | -8.7 | -9.0 | -7.4 |
| 88 | 442134 | Leptodactylone | Coumarin | -6.9 | -7.5 | -6.4 |
| 89 | 5280934 | linolenic acid | Linolenic acid | -6.5 | -6.7 | -5.6 |
| 90 | 530428 | Longiverbenone | Sesquiterpenoid | -4.9 | -8.1 | -7.3 |
| 91 | 5319116 | Luteolin 4'-O-β-D-glucuronopyranoside | Flavonoid | -9.7 | -10.0 | -8.5 |
| 92 | 13964550 | Luteolin 5-methyl ether | Flavonoid | -9.0 | -9.5 | -7.5 |
| 93 | 5318214 | Luteolin 7-methyl ether | Flavonoid | -9.8 | -9.8 | -7.8 |
| 94 |  | Luteolin 7-O-β-D-glucuronopyranoside | Flavonoid | -10.2 | -10.7 | -8.0 |
| 95 | 126962575 | Luteolin 7-O-β-D-glucuronopyranoside-6″-methyl ester | Flavonoid | -10.5 | -11.0 | -8.2 |
| 96 | 5280445 | Luteolin | Flavonoid | -9.8 | -9.6 | -7.9 |
| 97 |  | Methoxycyperotundol | Flavonoid | -6.6 | -8.1 | -6.7 |
| 98 | 288022 | Methyl 6-oxoheptanoate |  | -5.2 | -5.1 | -4.5 |
| 99 | 17399 | methyl 9-oxodecanoate |  | -5.4 | -5.2 | -4.6 |
| 100 | 14259 | methyl arachidate | Fatty acid | -5.5 | -6.6 | -5.0 |
| 101 | 5280590 | METHYL ELAIDATE | Fatty acid | -5.6 | -6.6 | -4.8 |
| 102 | 520298 | Methyl isotetradecanoate (iso-14-0) | Fatty acid | -5.9 | -5.9 | -4.9 |
| 103 | 8139 | methyl laurate | Fatty acid | -5.2 | -5.4 | -4.5 |
| 104 | 8181 | METHYL PALMITATE | Fatty acid | -5.5 | -6.0 | -5.0 |
| 105 | 8201 | Methyl stearate | Fatty acid | -5.3 | -6.4 | -5.0 |
| 106 | 31284 | Methyl tetradecanoate | Fatty acid | -5.4 | -5.7 | -4.8 |
| 107 | 12313013 | Mustakone | Sesquiterpenoid | -7.1 | -7.9 | -6.7 |
| 108 | 5491408 | Myricetin 3-O-β-D-galactopyranoside | Flavonoid | -8.8 | -10.7 | -8.0 |
| 109 | 5281672 | Myricetin | Flavone | -9.6 | -9.8 | -7.4 |
| 110 | 10582 | Myrtenol | (mono) Terpenoid | -5.5 | -6.0 | -5.5 |
| 111 | 129847769 | N-(1-deoxy-alpha-D-fructos-1-yl)-L-tryptophan | c-glycosyl | -8.0 | -9.2 | -6.8 |
| 112 | 610112 | N-(2-Acetylcyclopentylidene)cyclohexylamine |  | -7.1 | -7.1 | -6.5 |
| 113 | 439246 | Naringenin | Flavonoid | -9.0 | -9.4 | -7.8 |
| 114 | 50914217 | n-butyl-beta-D-fructopyranoside | Fructopyranoside | -4.7 | -6.2 | -4.6 |
| 115 | 24836956 | Neocurdione | Sesquiterpene | -7.5 | -8.3 | -6.7 |
| 116 | 610088 | N-Methyl-1-adamantaneacetamide | Amide group | -6.2 | -7.4 | -5.9 |
| 117 | 1268142 | NOOTKATONE | Sesquiterpenoid | -7.8 | -8.1 | -7.4 |
| 118 | 574096 | Octahydrobenzo[b]pyran, 4a-acetoxy-5,5,8a-trimethyl | Hydrocarbon | -4.5 | -6.0 | -5.0 |
| 119 | 445639 | Oleic acid | Fatty acid | -5.8 | -6.5 | -5.1 |
| 120 | 5281675 | Orientin | Flavonoid | -8.5 | -10.0 | -8.3 |
| 121 | 91692401 | Oxiraneundecanoic acid, 3-pentyl methyl ester, trans | Fatty acid | -5.5 | -6.5 | -5.1 |
| 122 | 484757 | Pallidol | Resveratrol | -7.6 | -5.3 | -8.5 |
| 123 | 985 | palmitic acid | Fatty acid | -5.6 | -5.9 | -4.8 |
| 124 | 445638 | palmitoleic acid | Fatty acid | -5.8 | -6.3 | -5.0 |
| 125 | 603359 | Phen-1,2-diol, 4-fluoro-5-aminoacetyl-, dimethyl ether | Ether | -6.0 | -6.3 | -5.6 |
| 126 | 6423450 | Phthalic acid, butyl undecyl ester | Acid | -6.7 | -7.3 | -6.1 |
| 127 | 191964 | Phthalic acid, di (2- propylpentyl) ester | A phthalate ester | -6.3 | -7.7 | -5.7 |
| 128 | 6423451 | Phthalic acid, isobutyl octadecyl ester | Aromatic dicarboxylic acid | -6.5 | -7.3 | -5.8 |
| 129 | 5280435 | Phytol | A diterpenoid and a long-chain primary fatty alcohol. | -6.7 | -6.8 | -5.6 |
| 130 | 667639 | Piceatannol | Polyphenol - stilbenoid | -8.6 | -8.7 | -7.0 |
| 131 | 5320651 | pogostol | Sesquiterpenoid | -7.3 | -8.2 | -6.7 |
| 132 | 3873459 | Prenyletin | Coumarin | -7.6 | -8.1 | -6.8 |
| 133 | 5748558 | Quercetin 7,3',4'-trimethyl ether | Flavonol | -9.3 | -9.4 | -7.7 |
| 134 | 5280343 | Quercetin | Flavone | -9.2 | -9.8 | -7.5 |
| 135 | 37439 | Quinic acid |  | -5.5 | -6.3 | -4.9 |
| 136 | 5283468 | rac-1-Monoolein | Fatty acid | -5.9 | -6.8 | -5.5 |
| 137 | 10634274 | Remirol | Benzofuranoid | -7.4 | -7.6 | -6.2 |
| 138 | 445154 | Resveratrol | Phytoalexin - polyphenols' stilbenoids group, | -8.0 | -8.1 | -6.6 |
| 139 | 5417 | rotundine | Alkaloid | -8.8 | -9.6 | -7.5 |
| 140 |  | Rotunduside F | Phenolic glycosides | -8.1 | -4.1 | -6.6 |
| 141 | 5280805 | Rutin | Flavonoid | -9.9 | -10.5 | -8.5 |
| 142 | 10801302 | Scabequinone | Benzofuranoid | -7.4 | -9.0 | -7.6 |
| 143 | 5280460 | Scopoletin | Coumarin | -6.8 | -7.4 | -6.3 |
| 144 | 92231 | Spathulenol | Sesquiterpenoid | -7.1 | -8.7 | -6.4 |
| 145 | 5281 | Stearic acid | Fatty acid | -5.6 | -6.5 | -4.8 |
| 146 | 5281295 | Sulfuretin | Flavonoid | -9.0 | -9.4 | -7.7 |
| 147 | 4916597 | Tetrahydrofuran-2-carboxylic acid, dibenzofuran-3-ylamide | Volatile organic compound | -8.5 | -9.5 | -7.7 |
| 148 | 5447 | Thiodiglycol | Organosulfur compound - polar protic solvent. | -3.4 | -3.6 | -2.9 |
| 149 | 91691549 | Trans-2-Dodecen-1-ol, trifluoroacetate | Fatty acid | -5.9 | -6.3 | -5.3 |
| 150 | 5281702 | Tricin | Flavonoid | -9.8 | -9.8 | -7.7 |
| 151 | 17453 | triphenylphosphoranylidene | Benzenamine | -5.2 | -6.7 | -7.4 |
| 152 | 5281426 | Umbelliferone | Coumarin | -6.8 | -7.0 | -6.4 |
| 153 | 75942 | Undecanoic acid,10-methyl | Fatty acid | -5.6 | -5.8 | -4.8 |
| 154 | 6029 | Uridine | Glycosylated pyrimidine | -6.3 | -7.2 | -5.3 |
| 155 | 9855795 | Valencene | Sesquiterpene | -7.1 | -7.7 | -7.5 |
| 156 | 8468 | Vanillic acid | Flavonoid | -5.8 | -5.9 | -5.7 |
| 157 | 11996452 | Viridiflorol | Sesquiterpenoid | -7.4 | -8.0 | -6.2 |
| 158 | 5280441 | Vitexin | Apigenin flavone glycosid | -8.7 | -11.0 | -7.4 |
| 159 | 5363633 | z-(13,14-Epoxy)tetradec-11-en-1-ol acetate | Fatty acid | -5.5 | -6.2 | -5.2 |
| 160 | 3973 | Ly294002 |  | -10.2 | -11.6 | -8.5 |
| 161 | 124193915 | 2,2-difluoroethyl (3S)-3-{[2'-amino-5-fluoro-2-(morpholin-4-yl)[4,5'-bipyrimidin]-6-yl]amino}-3-(hydroxymethyl)pyrrolidine-1-carboxylate |  | -9.3 |  |  |
| 162 | 53262401 | 3-[3-[4-(1-azanylcyclobutyl)phenyl]-5-phenyl-imidazo[4,5-b]pyridin-2-yl]pyridin-2-amine |  |  | -13.9 |  |
| 163 | 6022 | ADENOSINE-5'-DIPHOSPHATE |  |  |  | -7.0 |
| 164 | 25227436 | Capivasertib |  |  | -9.8 |  |
| 165 | 56649450 | Alpelisib |  | -10.0 |  |  |
|  |  |  |  |  |  |  |
