# Supplementary material for: Bioactivity of Cyperus amuricus extracts against hepatocellular carcinoma and molecular docking analysis targeting the PI3K/AKT/mTOR pathway
Source: PLoS One. 2026 Jan 9;21(1):e0340868. doi: 10.1371/journal.pone.0340868 (PMC12788648; doi:10.1371/journal.pone.0340868)
Supplement: S4 Table — (DOCX) [file pone.0340868.s005.docx]

Bioactivity of *Cyperus amuricus* Extracts Against Hepatocellular Carcinoma and Molecular Docking Analysis Targeting the PI3K/AKT/mTOR Pathway

**Thanh Luan Nguyen^1^, Thanh Khoi Tu^2,3^, Thien-Vy Phan^4^, Chanh M. Nguyen^5,6^ Khoa D. Nguyen^5,6^ Minh Quan Pham^7,8^,** **Hai Ha Pham Thi^2,3*^**

^1^ HUTECH Institute of Applied Science, HUTECH University, Ho Chi Minh City, Viet Nam

^2^ Center for Hi-Tech Development, Nguyen Tat Thanh University, Saigon Hi-Tech Park, Ho Chi Minh City, Vietnam.

^3^ NTT Hi-Tech Institute, Nguyen Tat Thanh University, Ho Chi Minh City, Vietnam.

^4^ Faculty of Pharmacy, Nguyen Tat Thanh University, Ho Chi Minh City, Vietnam

^5^ Institute of Applied Science and Technology, Van Lang School of Technology, Van Lang University, Ho Chi Minh City, Vietnam

^6^ Faculty of Applied Technology, Van Lang School of Technology, Van Lang University, Ho Chi Minh City, Vietnam

^7^ Institute of Natural Products Chemistry, Vietnam Academy of Science and Technology, Hanoi, Vietnam.

^8^ Graduate University of Science and Technology, Vietnam Academy of Science and Technology (VAST), Hanoi, Vietnam.

***** **Corresponding author:**

Email: [pthha@ntt.edu.vn](mailto:pthha@ntt.edu.vn) (Ph.D.)

**Short Title**

*Cyperus amuricus:* Anti-Hepatocellular Carcinoma and Molecular Docking Targeting the PI3K/AKT/mTOR Pathway

## Supporting information

**S4 Table. High binding affinity of *Cyperus amuricus*-derived compounds toward PI3K**

| **Code** | **Compounds** | **Class** | **∆G** |
| --- | --- | --- | --- |
|  |  |  | **(kcal/mol)** |
| 51 | Luteolin 7-O-β-D-glucuronopyranoside-6″-methyl ester | Flavonoids | -10,50 |
| 32 | 2,7-diphenyl-1,6-dioxopyridazino[4,5,2',3']pyrrolo[4',5'-d]pyridazine | Alkaloids | -10,30 |
| 50 | Luteolin 7-O-β-D-glucuronopyranoside | Flavonoids | -10,20 |
| 60 | Rutin | Flavonoids | -9,90 |
| 41 | Diosmetin | Flavonoids | -9,80 |
| 49 | Luteolin 7-methyl ether | Flavonoids | -9,80 |
| 52 | Luteolin | Flavonoids | -9,80 |
| 62 | Tricin | Flavonoids | -9,80 |
| 47 | Luteolin 4'-O-β-D-glucuronopyranoside | Flavonoids | -9,70 |
| 55 | Myricetin | Flavonoids | -9,60 |
| 116 | Estra-1,3,5(10)-trien-17β-ol | Steroids | -9,60 |
| 37 | Acacetin | Flavonoids | -9,40 |
| 38 | Apigenin | Flavonoids | -9,30 |
| 43 | Isorhamnetin | Flavonoids | -9,30 |
| 58 | Quercetin 7,3',4'-trimethyl ether | Flavonoids | -9,30 |
