# Supplementary material for: Bioactivity of Cyperus amuricus extracts against hepatocellular carcinoma and molecular docking analysis targeting the PI3K/AKT/mTOR pathway
Source: PLoS One. 2026 Jan 9;21(1):e0340868. doi: 10.1371/journal.pone.0340868 (PMC12788648; doi:10.1371/journal.pone.0340868)
Supplement: S5 Table — (DOCX) [file pone.0340868.s006.docx]

Bioactivity of *Cyperus amuricus* Extracts Against Hepatocellular Carcinoma and Molecular Docking Analysis Targeting the PI3K/AKT/mTOR Pathway

**Thanh Luan Nguyen^1^, Thanh Khoi Tu^2,3^, Thien-Vy Phan^4^, Chanh M. Nguyen^5,6^ Khoa D. Nguyen^5,6^ Minh Quan Pham^7,8^,** **Hai Ha Pham Thi^2,3*^**

^1^ HUTECH Institute of Applied Science, HUTECH University, Ho Chi Minh City, Viet Nam

^2^ Center for Hi-Tech Development, Nguyen Tat Thanh University, Saigon Hi-Tech Park, Ho Chi Minh City, Vietnam.

^3^ NTT Hi-Tech Institute, Nguyen Tat Thanh University, Ho Chi Minh City, Vietnam.

^4^ Faculty of Pharmacy, Nguyen Tat Thanh University, Ho Chi Minh City, Vietnam

^5^ Institute of Applied Science and Technology, Van Lang School of Technology, Van Lang University, Ho Chi Minh City, Vietnam

^6^ Faculty of Applied Technology, Van Lang School of Technology, Van Lang University, Ho Chi Minh City, Vietnam

^7^ Institute of Natural Products Chemistry, Vietnam Academy of Science and Technology, Hanoi, Vietnam.

^8^ Graduate University of Science and Technology, Vietnam Academy of Science and Technology (VAST), Hanoi, Vietnam.

***** **Corresponding author:**

Email: [pthha@ntt.edu.vn](mailto:pthha@ntt.edu.vn) (Ph.D.)

**Short Title**

*Cyperus amuricus:* Anti-Hepatocellular Carcinoma and Molecular Docking Targeting the PI3K/AKT/mTOR Pathway

## Supporting information

**S5 Table. High binding affinity of *Cyperus amuricus*-derived compounds toward AKT**

| **Number** | **Compounds** | **Class** | **∆G** |
| --- | --- | --- | --- |
|  |  |  | **(kcal/mol)** |
| 51 | Luteolin 7-O-β-D-glucuronopyranoside-6″-methyl ester | Flavonoids | -11,00 |
| 64 | Vitexin | Flavone glycoside | -11,00 |
| 50 | Luteolin 7-O-β-D-glucuronopyranoside | Flavonoids | -10,70 |
| 54 | Myricetin 3-O-β-D-galactopyranoside | Flavonoids | -10,70 |
| 36 | 7,3’-dihydroxy-5,5’-dimethoxy-8-prenylflavan | Flavonoids | -10,60 |
| 42 | Epiorientin | Flavonoids | -10,60 |
| 60 | Rutin | Flavonoids | -10,50 |
| 34 | 5,7,3’-trihydroxy-5’-methoxy-8-prenylflavan | Flavonoids | -10,20 |
| 47 | Luteolin 4'-O-β-D-glucuronopyranoside | Flavonoids | -10,00 |
| 57 | Orientin | Flavonoids | -10,00 |
