# Supplementary material for: Bioactivity of Cyperus amuricus extracts against hepatocellular carcinoma and molecular docking analysis targeting the PI3K/AKT/mTOR pathway
Source: PLoS One. 2026 Jan 9;21(1):e0340868. doi: 10.1371/journal.pone.0340868 (PMC12788648; doi:10.1371/journal.pone.0340868)
Supplement: S7 Table — (DOCX) [file pone.0340868.s008.docx]

Bioactivity of *Cyperus amuricus* Extracts Against Hepatocellular Carcinoma and Molecular Docking Analysis Targeting the PI3K/AKT/mTOR Pathway

**Thanh Luan Nguyen^1^, Thanh Khoi Tu^2,3^, Thien-Vy Phan^4^, Chanh M. Nguyen^5,6^ Khoa D. Nguyen^5,6^ Minh Quan Pham^7,8^,** **Hai Ha Pham Thi^2,3*^**

^1^ HUTECH Institute of Applied Science, HUTECH University, Ho Chi Minh City, Viet Nam

^2^ Center for Hi-Tech Development, Nguyen Tat Thanh University, Saigon Hi-Tech Park, Ho Chi Minh City, Vietnam.

^3^ NTT Hi-Tech Institute, Nguyen Tat Thanh University, Ho Chi Minh City, Vietnam.

^4^ Faculty of Pharmacy, Nguyen Tat Thanh University, Ho Chi Minh City, Vietnam

^5^ Institute of Applied Science and Technology, Van Lang School of Technology, Van Lang University, Ho Chi Minh City, Vietnam

^6^ Faculty of Applied Technology, Van Lang School of Technology, Van Lang University, Ho Chi Minh City, Vietnam

^7^ Institute of Natural Products Chemistry, Vietnam Academy of Science and Technology, Hanoi, Vietnam.

^8^ Graduate University of Science and Technology, Vietnam Academy of Science and Technology (VAST), Hanoi, Vietnam.

***** **Corresponding author:**

Email: [pthha@ntt.edu.vn](mailto:pthha@ntt.edu.vn) (Ph.D.)

**Short Title**

*Cyperus amuricus:* Anti-Hepatocellular Carcinoma and Molecular Docking Targeting the PI3K/AKT/mTOR Pathway

## Supporting information

**S7 Table. Molecular interactions of Ly294002 with PI3K, AKT, and mTOR proteins**

| **Protein** | **Types of interactions** | **Amino acid residues** |
| --- | --- | --- |
| PI3K | Hydrogen bond | Val851, Ser854 |
|  | π-Alkyl hydrophobic | Ile800, Val850 |
|  | π-σ hydrophobic | Ile848, Ile932 |
|  | π-π stacking | Tyr836 |
|  | π-sulfur bond | Met922 |
|  | Carbon-hydrogen bond  (C–H⋯O) | Val271, Tyr272, Gln79 |
| AKT | π-Alkyl hydrophobic | Leu210 |
|  | π-σ hydrophobic | Val270 |
|  | π-π stacking | Trp80 |
| mTOR | Hydrogen bond | Thr2245 |
|  | π-Alkyl hydrophobic | Pro2169 |
|  | π-σ hydrophobic | Leu2185, Met2345 |
|  | π-π stacking | Trp2239 |
